# He–Ne laser accelerates seed germination by modulating growth hormones and reprogramming metabolism in brinjal

**DOI:** 10.1038/s41598-021-86984-8

**Published:** 2021-04-12

**Authors:** Puthanvila Surendrababu Swathy, Kodsara Ramachandra Kiran, Manjunath B Joshi, Krishna Kishore Mahato, Annamalai Muthusamy

**Affiliations:** 1 Department of Plant Sciences, Manipal School of Life Sciences, Manipal Academy of Higher Education, Manipal, Karnataka 576104 India; 2 Department of Ageing Research, Manipal School of Life Sciences, Manipal Academy of Higher Education, Manipal, Karnataka 576104 India; 3 Department of Biophysics, Manipal School of Life Sciences, Manipal Academy of Higher Education, Manipal, Karnataka 576104 India

**Keywords:** Plant sciences, Plant molecular biology, Plant physiology

## Abstract

A plant’s ability to maximize seed germination, growth, and photosynthetic productivity depends on its aptitude to sense, evaluate, and respond to the quality, quantity, and direction of the light. Among diverse colors of light possessing different wavelengths and red light shown to have a high impact on the photosynthetic and growth responses of the plants. The use of artificial light sources where the quality, intensity, and duration of exposure can be controlled would be an efficient method to increase the efficiency of the crop plants. The coherent, collimated, and monochromatic properties of laser light sources enabled as biostimulator compared to the normal light. The present study was attempted to use the potential role of the He–Ne laser as a bio-stimulator device to improve the germination and growth of brinjal and to investigate the possible interactions of plant and laser photons. A substantial enhancement was observed in germination index, germination time and seed vigor index of laser-irradiated than control groups. The enhanced germination rate was correlated with higher GA content and its biosynthetic genes whereas decreased ABA content and its catabolic genes and GA/ABA ratio were noted in laser-irradiated groups during seed germination than control groups. Further the expression of phytochrome gene transcripts, *PhyA* and *PhyB1* were upregulated in laser-irradiated seedlings which correlate with enhanced seed germination than control. Elevated levels of primary metabolites were noted in the early stages of germination whereas modulation of secondary metabolites was observed in later growth. Consequently, significantly increased photosynthetic rate, stomatal conductance, and transpiration rate was perceived in laser-irradiated seedlings compare with control. The current study showed hormone and phytochrome-mediated mechanisms of seed germination in laser-irradiated groups along with the enhanced photosynthetic rate, primary and secondary metabolites.

## Introduction

He–Ne laser is being used as low energy and continuous irradiation in the field of agriculture and considered as an environment-friendly method for the enhancement of plant growth, agronomical, and stress resistance traits^1^. The low-level laser irradiation acts as a bio-stimulator on the biological system and it is a proven strategy to enhance seed germination, vegetative mass, photosynthetic system and increment in the crop yield. In a nutshell, the prospective application of He–Ne laser during crop cultivation demonstrated the enhanced seed germination^2,3^, seed energy potential^4^, positive impact on seed vigor^5,6^, seedling elongation^7^, an increment of photosynthetic pigment content^2^, in vivo and in vitro growth, agronomical characters^4,6,8,9^ and a hindrance to diseases and abiotic stress^10^. Red laser exhibited a repairing role by enhancing the physiological characters in the plants exposed to various abiotic stress such as drought^9^, UV-B induced stress^7,10–15^, and salinity stress^16,17^ and substantial biochemical changes such as higher phenolic content, soluble sugar, total soluble content, protein and nitrogen contents in triticale seeds^18^, *Lagenaria*^19^, α-amylase, and protease activities in brinjal^2^, sunflower^20,21^, safflower^22^, *Erigeron*^23^, barley^24^, cucumber^25,26^ was noted due to low-level laser irradiation.

Seed germination is a complex process controlled by two plant hormones such as Abscisic acid (ABA) and Gibberellic acid (GA). ABA sustains seed dormancy whereas seed germination is accelerated by GA^27^. During a favorable environment, the degradation of ABA and intensification of GA biosynthesis releases the dormancy in seeds^28^. Phytochrome, the extensively studied photoreceptors in the plant system perceive red and far-red light and play fundamental roles in seed germination, seedling de-etiolation^29^, shade avoidance^30^, and flowering^31^. Five distinct phytochromes were identified in the tomato family in which *PhyA, Phy1*, *PhyB2*, and *PhyE* are classified as Phy subfamilies and *PhyF* is designated as a new subfamily^32^. The differential expression of phytochrome transcripts such as *phyA, phyB1, phyB2, phyAB1*, and *phyAB1B2* was observed and the expression of *phyB2* was noted under red or far-red light^29,33^.

Previous studies have shown that light-mediated seed germination is primarily regulated by phytochrome B, followed by phytochrome A. It has a major impact on seed germination by controlling two major plant hormones, ABA and GA, which are regulated antagonistically. The synergistic effect of a laser beam and phytochrome receptors formed the basis of the stimulatory process in seed germination and subsequent growth of seedlings^34^. As metabolites are end and functional products responsible to regulate physiological processes, understanding metabolome in a given condition might be relevant. Also, the un-targeted metabolite profiling of plants can provide the information of stimuli-induced dynamics of metabolites, identification and quantification. In our earlier investigation, we have reported the improved seed germination and increment of photosynthesis pigment content^3^, in vivo and in vitro growth and agronomical characters^7,9^ with laser irradiation in *Solanum melongena* (var Mattu Gulla) and *Withania somnifera*^9^. The *Solanum* belongs to the Solanaceae family, which consists of 450 species and has largest monophyletic group. Currently, chromosome-anchored genome sequences were reported in several genera of the Solanaceae family such as potato, tomato and pepper^35–37^. Mattu Gulla is a unique and indigenous variety of brinjal with Geographical Indication (GI) status, which is being cultivated in Mattu village, Udupi District, India. Based on our earlier observations^2,6,8^ in brinjal and existing literature in other crops, it is very clear that the laser treatment enhances seed germination, growth and agronomical characters. However, the molecular mechanism associated with enhanced seed germination is remained not known. Thus the present study is hypothesized that laser induces a beneficial effect on seed germination and subsequent developmental stages through phytochrome-phytohormone-mediated signal transduction and by altering the metabolomics of the seedlings. Further, the study helps in deciphering the molecular events associated with germination mediated by laser irradiation, which helps to use He–Ne laser as a potential tool to break dormancy, enhance germination and growth of the plants. Therefore, the present study aimed to understand the hormonal- and phytochrome-mediated seed germination and associated metabolic changes in response to He–Ne laser irradiation in brinjal var. Mattu Gulla.

## Results

### Laser-irradiation modulated hormones profile

**Figure 1:**
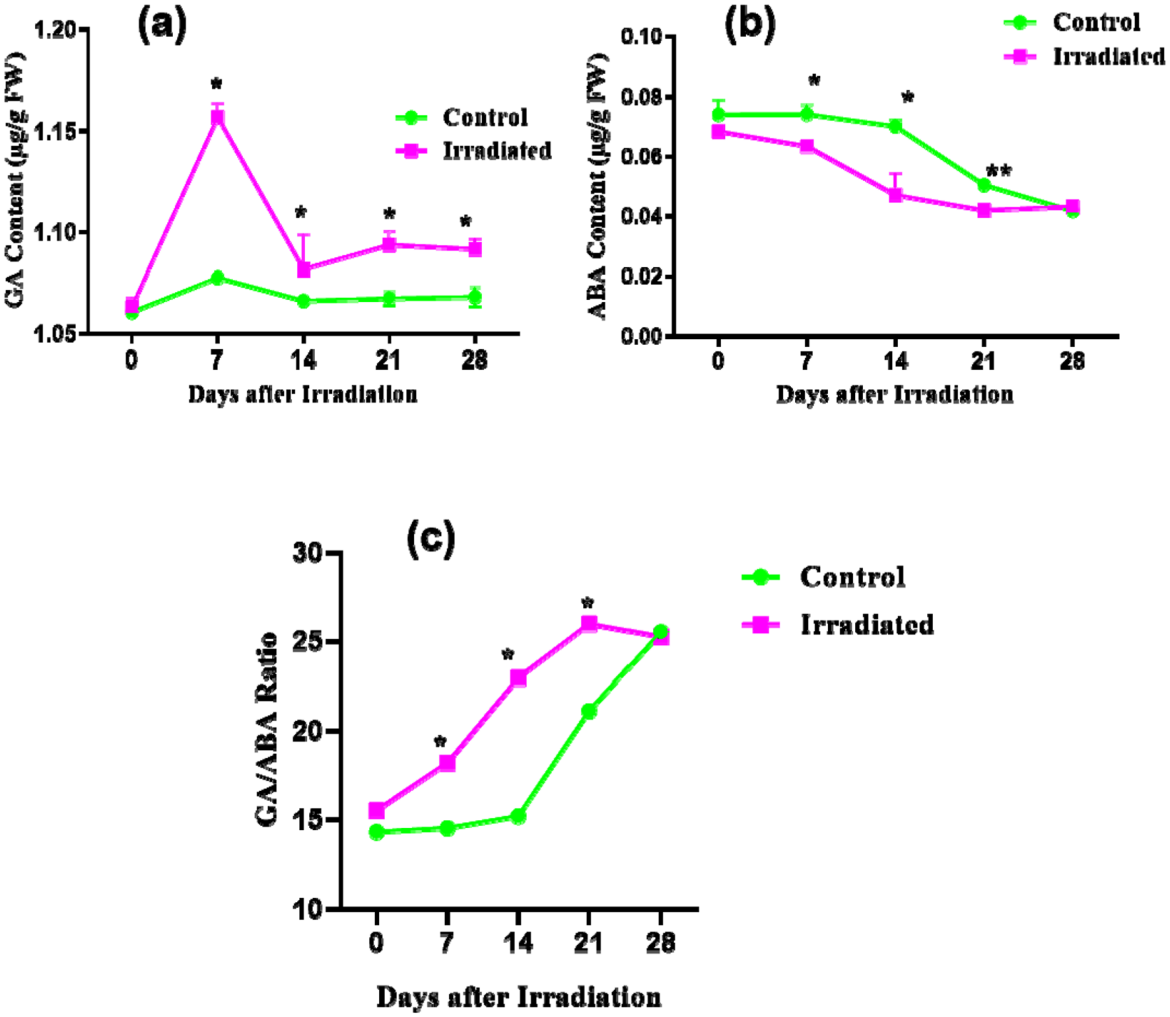
Quantification of phytohormones from control and laser-irradiated seeds and seedlings (**a**) gibberellic acid, (**b**) abscisic acid and (**c**) GA/ABA ratio from seeds and seedlings of brinjal (*Solanum melongena* L.) var. Mattu Gulla in response to He–Ne laser irradiation and un-irradiated control during different developmental stages (0–28 DAI). Data are expressed as mean ± SD and significant at **p < 0.01 and *p < 0.05 compared with non-irradiated control (n = 2).

Quantitative estimation of GA and ABA from the control and laser-irradiated group (25 J/cm^2^) was performed during different days after irradiation (DAI). The GA and ABA were quantified in seeds until germination (0 and 7 days after irradiation) and later in leaves during the developmental stages (14, 21, and 28 days after irradiation), and compared among control and laser-irradiated groups. Among all the developmental stages, the highest GA content was observed at 7 DAI. The laser-irradiated germinating seeds showed a significant increase in GA content at 7 DAI with a fold change of 0.8 (*p < 0.05). The GA content was gradually decreased in later stages of seed germination. Twenty-one-day-old seedlings from laser-irradiated seeds showed a 0.024 (*p < 0.05) fold increase in the GA content than the control group. A similar development was observed in 28-day old seedlings from laser irradiated seeds with a fold change of 0.022 (*p < 0.05) in comparison with un-irradiated control (Fig. 1a). The ABA content was higher in 0 DAI control seeds than the laser-irradiated seeds with a fold change of 0.08. A gradual decline in the ABA content was observed in the following days of germination and seedling development. The laser-irradiated seeds showed a lower ABA content than the control with a fold change of − 0.14 (*p < 0.05) at 7 DAI. Further, a significant change was observed in the 14 and 21 DAI with a fold change of − 0.33 (*p < 0.05) and − 0.17 (**p < 0.01) respectively in the laser-irradiated group than control with high ABA content. The ABA level was very lower in the 28 DAI in both the experimental groups (Fig. 1b). The GA/ABA ratio is critical for the seeds to break dormancy and entering the germination phase. The GA/ABA ratio was comparatively higher in laser-irradiated groups with a substantial increase over the growth phase. The highest GA/ABA ratio was detected at 21 DAI (*p < 0.05) and lowest at the 0 DAI (Fig. 1c).

### Quantitative real-time PCR analysis of hormones and phytochrome transcripts

#### Expression levels of gibberellic acid synthesis genes

**Figure 2:**
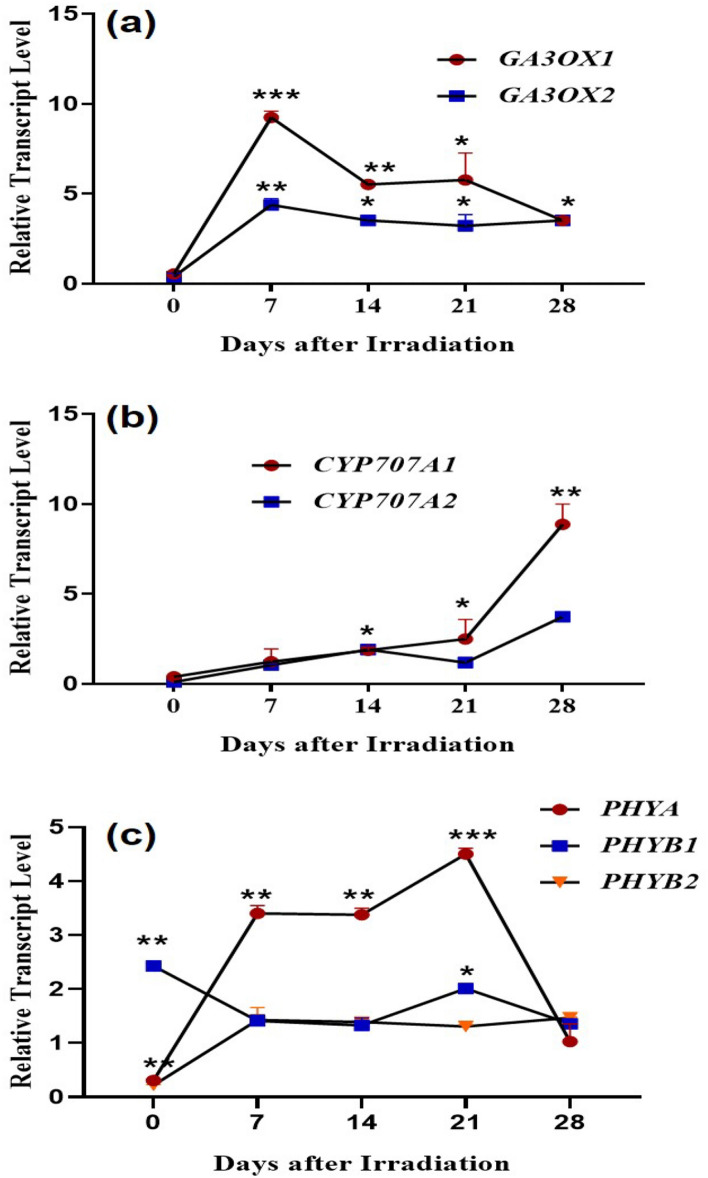
Expression of the genes involved in (**a**) gibberellic acid biosynthesis (*GA3ox1* and *2*) (**b**) ABA inactivation (*CYP707A1* and *–2*) and (**c**) phytochrome regulation (*PHYA, B1* and *B2*) from seeds and seedlings of brinjal (*Solanum melongena* L.) var. Mattu Gulla in response to He–Ne laser irradiation and un-irradiated control during 0–28 DAI. The transcript level represented are normalized to 18S rRNA and significant at ***p < 0.001, **p < 0.01, and *p < 0.05 compared with un-irradiated control (n = 2).

The GA is an integral factor for germination, and evaluating the transcript levels of genes encoding the bioactive GA is important to understand its involvement in the enhanced seed germination. The final phase of GA biosynthesis is catalyzed by GA 3-oxidase (GA3ox1 and GA3ox2). The expression level of *GA3ox1* at 0 DAI was slightly lower in laser-irradiated seeds in comparison with un-irradiated control. The expression level of *Ga3ox1* was highly upregulated with a fold change of 8.24 (***p < 0.001), 4.52 (**p < 0.01), 4.77 (*p < 0.05) in laser-irradiated groups at 7, 14 and 21 DAI respectively than the control group (Fig. 2a). The expression pattern of *GA3ox2* was similar to *GA3ox1*. The expression of *GA3ox2* was marginally downregulated in the laser-irradiated seeds than the control with a fold change of − 0. 61 at 0 DAI. A drastic upregulation of *GA3ox2* was observed in the laser-irradiated group at 7 DAI, with a fold increase of 4.37 (**p < 0.01). During developmental stages, the expression was higher in the laser-irradiated group in comparison with the control group with a fold change of 2.52, 2.22, and 2.51 at 14, 21, 28 DAI respectively (*p < 0.05). However, the expression remained constant in the laser-irradiated group (Fig. 2a).

#### Expression levels of abscisic acid catabolic genes

To validate the endogenous level of ABA in the control and laser-irradiated groups, qRT-PCR was performed and evaluated the transcript level of ABA catabolic genes *CYP707A1-A2*. The expression of *CYP707A1* was gradually increased over the development in the laser-irradiated group. Relative to its expression on the 0 DAI, the *CYP707A1* level was higher in 7 DAI, with a fold change of 2.13 in laser-irradiated seeds. During 14 and 21 DAI, the expression was slightly upregulated in laser-irradiated seedlings than the control group with a fold change of 0.86 and 1.45 respectively. The highest expression of *CYP707A1* was observed in the 28 DAI, indicating the lowest endogenous content in laser-irradiated seedlings than control with a fold change of 7.87 (**p < 0.01) (Fig. 2b). The expression pattern of *CYP707A2* was similar to *CYP707A1* except in 21 DAI with downregulation of expression than 14 DAI. As similar to *CYP707A1*, the highest level of expression of *CYP707A2* was observed in the 28 DAI (*p < 0.05) than all the other experimental groups (Fig. 2b).

#### Expression levels of phytochrome regulating genes

The phytochrome regulation on seed germination and seedling development was evaluated during 0–28 DAI using qRT- PCR. Initially, the transcript level of *PhyA* in laser-irradiated seeds was lower than the control with a fold change of − 0.7 and the expression level was highly upregulated in laser-irradiated seedlings during the later stage of development (Fig. 2c). The *PhyB1* expression is found to be influenced by the He–Ne laser irradiation since the transcript level was upregulated at 0 DAI than control with a fold change of 1.42 (*p < 0.01). The comparative analysis of *PhyB1* transcript level of the laser-irradiated group from 7 to 28 DAI, indicated the highest expression during 21 DAI with a fold change of 1.00 (*p < 0.05) (Fig. 2c). The laser-irradiated seeds not only showed significant downregulation of *PhyB2* level (***p < 0.001) during the initial hours after irradiation, and also revealing a similar level of *PHYB2* expression in the following development stage. Subsequently, the expression of *PhyB2* was marginally upregulated during different time points in laser-irradiated seeds (Fig. 2c), indicating the non-involvement of *PhyB2* in a laser enhanced germination and seedling development.

### Seed and leaf metabolite profiling

**Figure 3:**
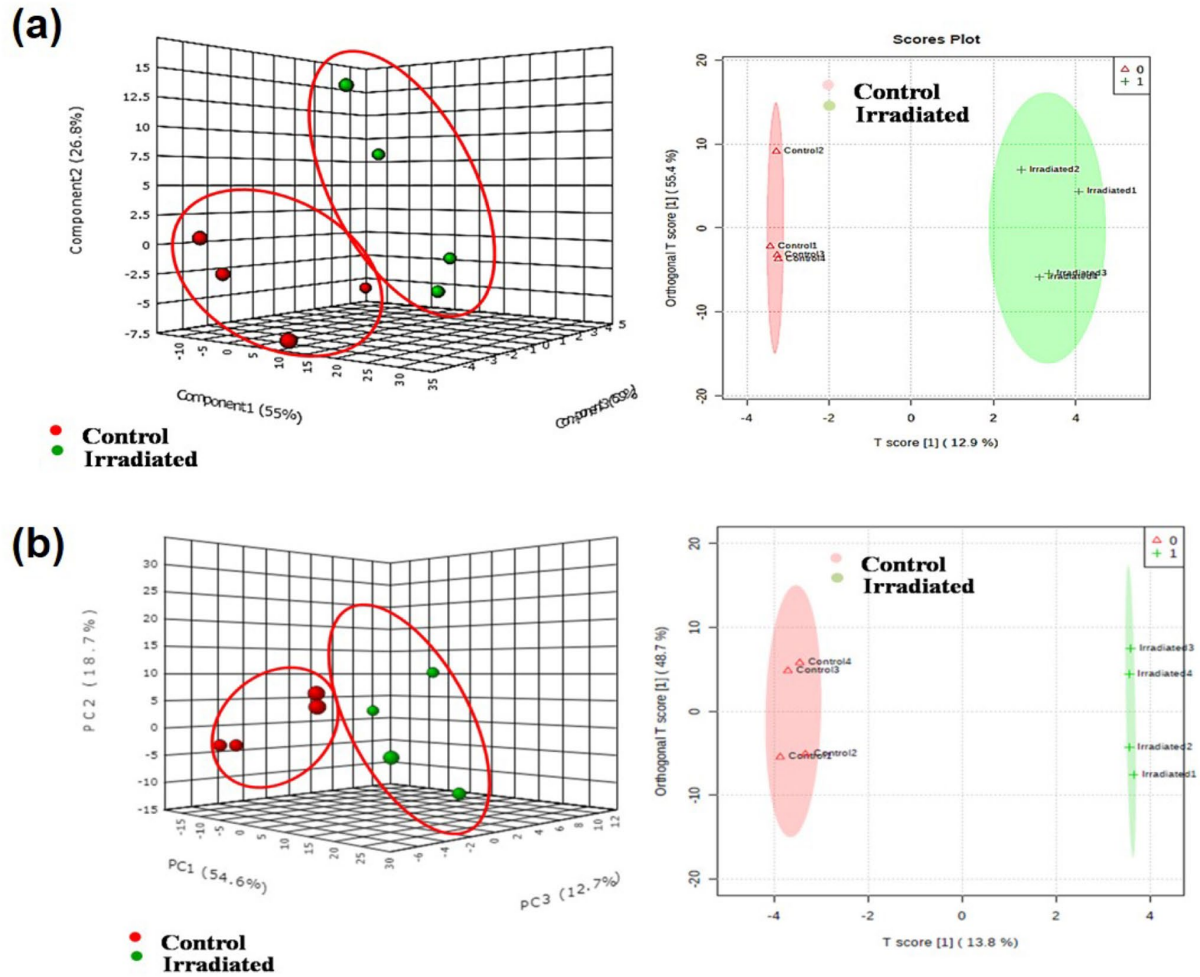
Multivariate analysis score plots of metabolites of seeds principal component analysis (PCA) and orthogonal partial least squares discriminant analysis (OPLS-DA) score plot of control and laser-irradiated group at (**a**) 1 DAI and (**b**) 7 DAI. Data were obtained from positive and negative ESI ionization mode (n = 2).

**Figure 4:**
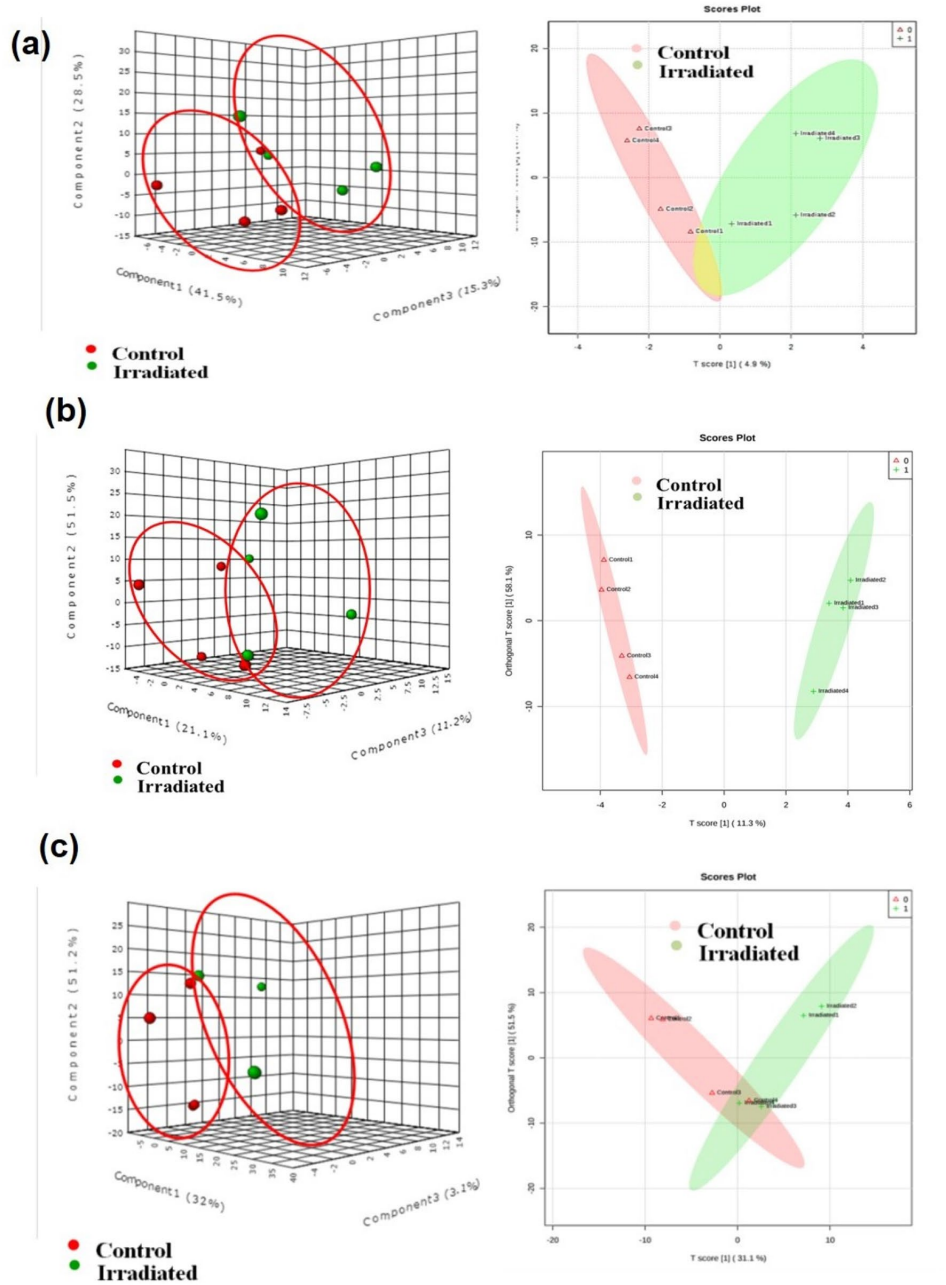
Multivariate analysis score plots of metabolites of seedlings Principal component analysis (PCA) and Orthogonal partial least squares discriminant analysis (OPLS-DA) score plot of control and laser-irradiated group at (**a**) 14 DAI (**b**) 21 DAI and (**c**) 28 DAI. Data were obtained from positive and negative ESI ionization mode (n = 2).

An in-house liquid chromatography-Quadrapole-time of flight-Mass spectrometry platform was used to perform untargeted metabolomics of seeds and leaf of laser-irradiated and control groups with the same time interval. The aligned and normalized metabolite profiling data were subjected to principal component analysis (PCA) and orthogonal partial least squares-discriminant analysis (OPLS-DA) by Metaboanalyst software. Since the experimental groups were independent, the PCA score plot was aggregated into groups and a clear distinction of the samples was noted for 1, 7 (Fig. 3a, b), 14, 21 and 28 DAI (Fig. 4a–c) respectively. Further, OPLS-DA, a tool used for dimension reduction and identification of spectral features for group separation was applied. The OPLS-DA data indicated the separation of control and laser-irradiated seeds in positive and negative modes for 1, 7 (Fig. 3a, b), 14, 21 and 28 DAI (Fig. 4a–c), except an overlap was observed in 14 and 28 DAI. The two different clusters in each group representing the positive and negative mode samples delineated through the multivariate analysis. The plot thus indicates the substantial variations in the composition of metabolites during the seed to the seedling establishment in control and laser-irradiated groups.

### Identification and quantification of primary metabolites

The spectral features present in 50% of the samples from each experimental group were subjected to metabolite identification and the mass was provided with a tolerance of ± 30 ppm. The identified metabolites were mainly classified into primary and secondary metabolites. Further, the primary metabolites were classified into amino acids, sugars, carbohydrates, organic acids and fatty acids. Similarly, the secondary metabolites were also classified into alkaloids, terpenoids, flavonoids, lignans and hormone derivatives in all the experimental groups (Tables S1 and S2). The most predominant bioactive molecules such as amino acids and their derivatives were detected and quantified in the study (Figure S2). A total of 12 amino acids were identified and found to be different in the abundance due to laser irradiation after 24 h incubation (1 DAI). Alanine and aspartate (*p < 0.05), which are synthesized by the transamination of oxaloacetate and pyruvate were significantly higher in the irradiated seeds than in control. The abundance level of branched-chain amino acids, leucine/isoleucine (***p < 0.001) and valine (**p < 0.01) were comparatively higher upon laser irradiation. Shikimate derived phenylalanine (*p < 0.05), tryptophan (**p < 0.01) and tyrosine (*p < 0.05) levels were increased in the laser-irradiated seeds in comparison to un-irradiated control. In contrast, glutamate and aspargine were reduced in the seeds which were irradiated by low-level laser (Figure S2a). In 7 DAI seeds, 13 amino acids were identified, in which most of the amino acids such as arginine (***p < 0.001), glutamine (***p < 0.001), alanine (**p < 0.01), tryptophan (*p < 0.05), asparagine (*p < 0.05) and leucine and isoleucine (*p < 0.05) were significantly increased in the laser-irradiated groups in comparison with un-irradiated control. The gamma-aminobutyric acid (GABA) was exhibited higher abundance during the early stages of germination in the laser-irradiated group (Figure S2b). The amino acid abundance continuously increased over the early seedling stage (14 DAI) in the laser-irradiated group. Out of 10 amino acids identified, pyruvate-derived leucine/isoleucine was reduced considerably, whereas GABA, arginine, asparagine, glutamine, proline, phenylalanine, threonine and valine showed a boost in abundance (Figure S2c). The 21 DAI group, exhibited a reduction in the amino acid abundance, such as glutamine, leucine/isoleucine and phenylalanine. The enhancement in abundance was noted in valine (*p < 0.05) (Figure S2d) in 21 DAI. The 28 DAI fully developed seedling showed a higher abundance of pyruvate derived alanine (*p < 0.05) and valine (***p < 0.001), glutamate derived arginine (*p < 0.05), and glutamine (**p < 0.01), whereas leucine/isoleucine content was found to be lower in the laser-irradiated group compared to control (Figure S2e).

The sugar abundance level at 1 DAI indicated that the sugar metabolism was increased significantly upon laser irradiation. Sucrose, the key intermediate involved in the carbohydrate metabolism in seeds were found to be considerably higher (**p < 0.01) in the laser-irradiated groups. The sucrose-derived glucose/galactose (*p < 0.05) were also found to be higher in abundance along with ribose, galactose and raffinose (*p < 0.05) (Figure S3a). The sugar level exhibited the same pattern in 7 DAI seeds, whereas glucose and sucrose showed a significant upregulation in the laser-irradiated group compared to the control. Galactitol, a reduction product of galactose was found to be decreased in germinated seeds in response to laser irradiation (Figure S3b). The glucose, galactose, fructose, myo-inositol levels were increased in the 14 DAI seedlings (Figure S3c). Sucrose, maltose, and trehalose and sucrose-derived monosaccharides such as glucose-6-phosphate were high in 21 DAI seedlings (Figure S3d). Furthermore, the glucose, galactose, fructose, xylose, sucrose, trehalose, and myo-inositol were increased in the laser-irradiated groups during the final stage of seedling development (28 DAI). In contrast, the abundance level of ribose was found to be lower in the 28 DAI seedlings compared to un-irradiated control (Figure S3e). The organic acids and the intermediates of TCA cycles such as succinate, fumarate, citrate/isocitrate, and malate, the most predominant and essential fatty acids are described in Supplementary Information (Figure S4a–e).

### Identification and quantification of secondary metabolites

**Figure 5:**
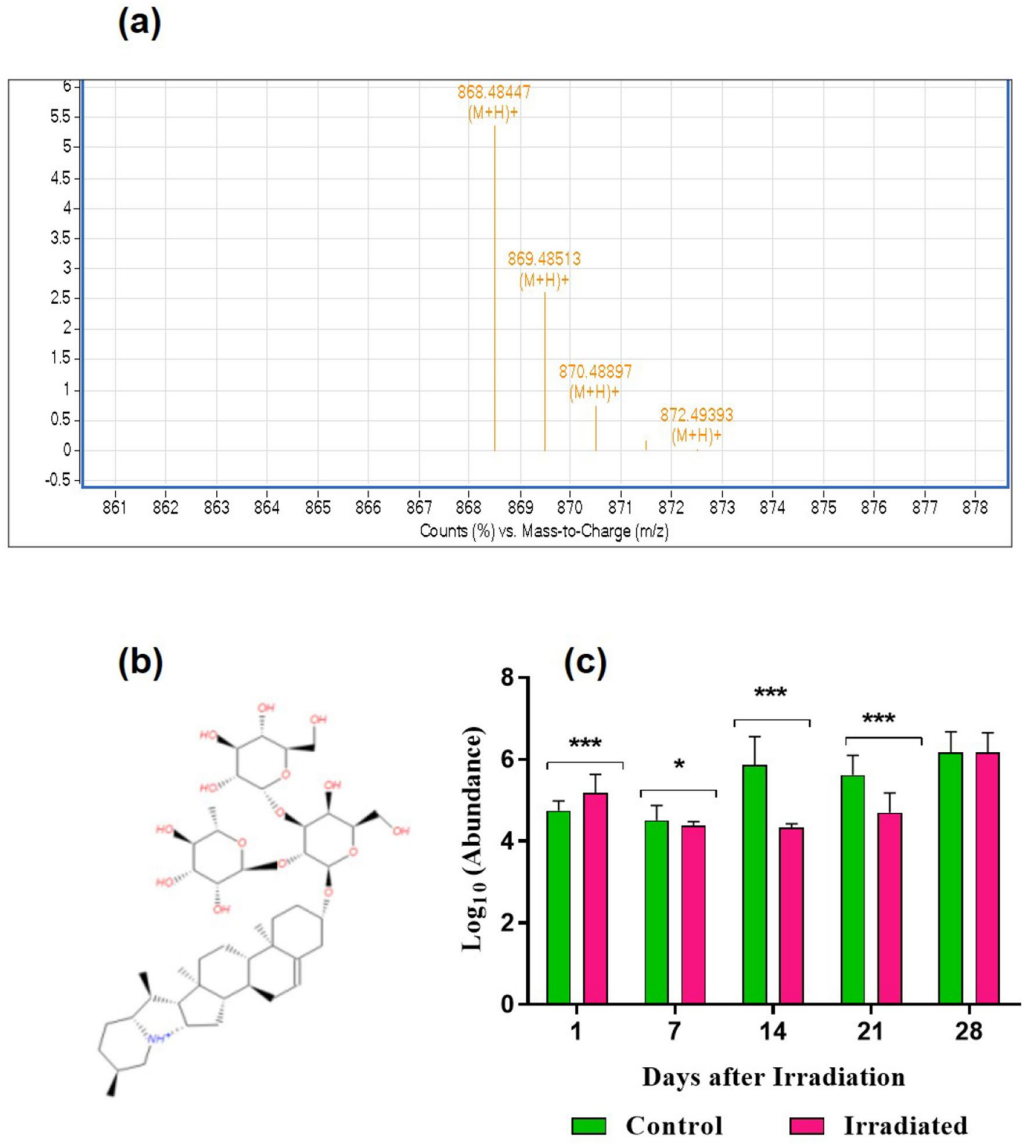
Detection and quantification of α-solanine from seeds and seedlings of brinjal (*Solanum melongena* L.) var. Mattu Gulla in response to He–Ne laser irradiation and un-irradiated control. (**a**) HPLC–ESI–MS base peak chromatogram of α-solanine, (**b**) structure and (**c**) Abundance value (log10) obtained from the control and laser-irradiated group. Statistically significant changes in metabolite intensity between the control and laser-irradiated groups are represented as ***p < 0.001 and *p < 0.05 (n = 2).

**Figure 6:**
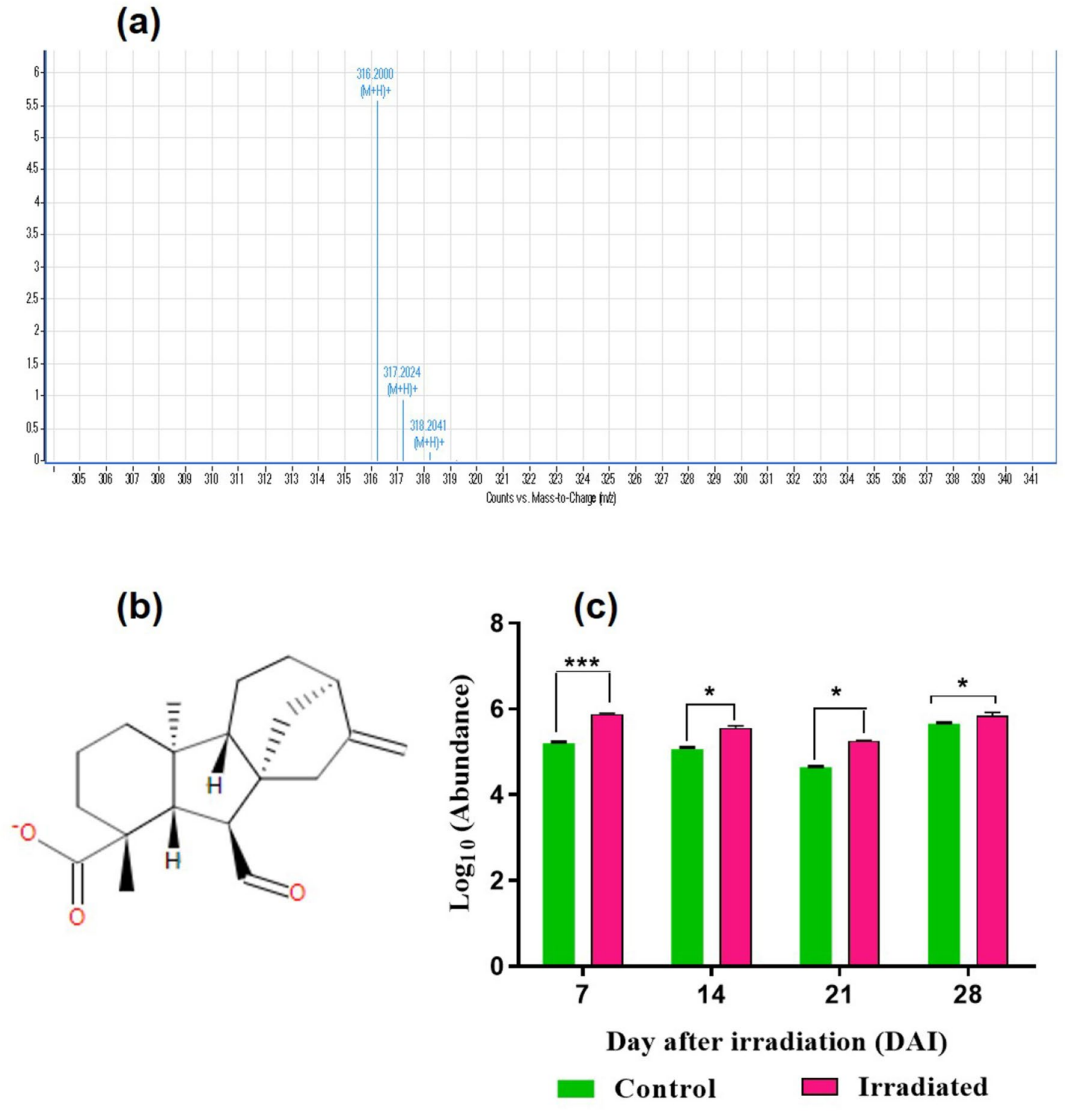
Detection and quantification of GA12-aldehyde from seeds and seedlings of brinjal (*Solanum melongena* L.) var. Mattu Gulla in response to He–Ne laser irradiation and un-irradiated control. (**a**) HPLC–ESI–MS base peak chromatogram of GA12-aldehyde, (**b**) structure and (**c**) abundance value (log10) from the control and laser-irradiated group. Statistically significant changes in metabolite intensity between the control and laser-irradiated groups are represented as ***p < 0.001 and *p < 0.05 (n = 2).

A substantial alteration in various secondary metabolites was noted in the present study. Glycoalkaloids are a class of nitrogen-containing steroidal glycosides commonly found in the Solanaceae family. The study was able to detect the most predominant glycoalkaloids, α-solanine (m/z-868.5058457121) throughout the developmental stages (Fig. 5a–c). The abundance was higher in laser-irradiated seeds after 24 h incubation (1 DAI) in comparison with un-irradiated control. The level of α-solanine was gradually decreasing in the following developmental stages including 7 (*p < 0.05), 14 (**p < 0.01) and 21 DAI (***p < 0.001), and the abundance was found to be similar and non-significant during seedling development (Fig. 5c). GA and ABA are synthesized from isoprene subunits via isoprenoid pathways in the chloroplasts and cytosol. In the present study, Gibberellin A12-aldehyde a precursor of GA_12_ biosynthesis was detected from 7 to 28 DAI with an m/z value of 316.20 (Fig. 6a–c). GA_12_ is the branch point gibberellic acid in plants, to form the whole spectrum of GA through oxidations and cyclizations. In the 7 DAI, a highly significant abundance of GA_12_ was observed in the laser-irradiated seeds than control with a fold change of 0.13 (***p < 0.001) and found consistently higher in the irradiated seeds with a fold change of 0.10,0.13 and 0.03 respectively (*p < 0.05) (Fig. 6c) in 14–28 DAI. The abundance was found to be higher in the laser-irradiated tissues over control. The unique metabolites identified in laser-irradiated seeds and seedlings were depicted in Table S1. Among the identified, the most noted metabolites in laser-irradiated groups are 7′-hydroxyabscisate (7-HA) which was recorded on 1 DAI, and it is involved in the ABA degradation in several plants. Furthermore, this compound was known to be tangled in the cellular homeostasis of ABA, which is particularly synthesized in the plants via the de novo pathway.

### The metabolic pathway analysis

**Figure 7:**
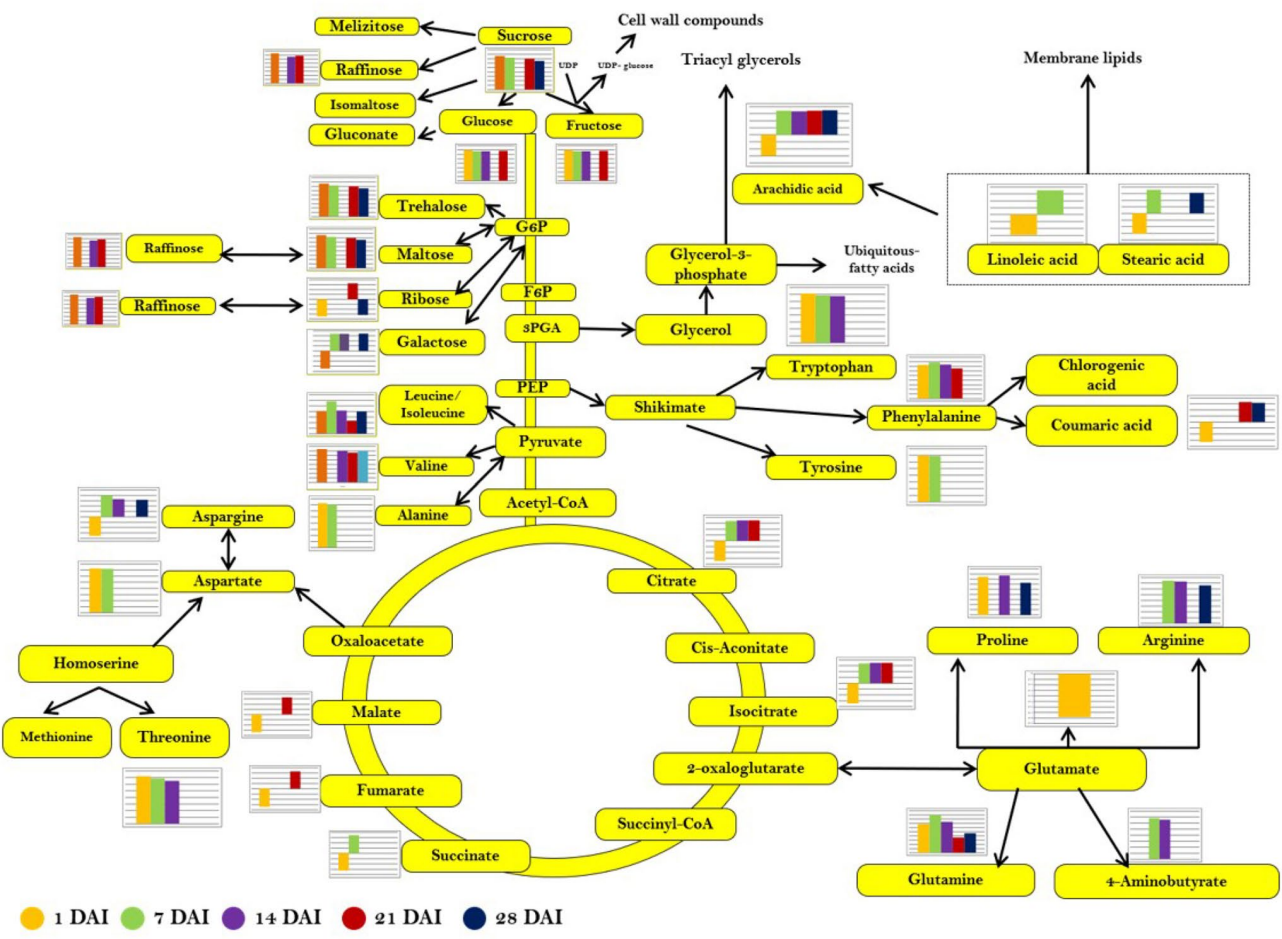
Comprehensive metabolic map of primary metabolites and associated secondary metabolites detected in the study during different developmental stages in laser-irradiated seeds and seedlings (1–28 DAI).

The pathway analysis was carried out using Metaboanalyst (MetPA) with the identified metabolites from each developmental stage to identify the most relevant metabolic pathways involved in response to laser irradiation. As illustrated in Figure S4a–e, the reconstruction of the metabolic pathways with the altered metabolites in 1 DAI results in changes in 21 pathways. The most predominant pathways to be altered was aminoacyl t-RNA biosynthesis [− log (*p*) 1.68], alanine, aspartate and glutamate metabolism [− log (*p*) 1.28], starch and sucrose metabolism [− log (*p*) 6.10], TCA cycle [− log (*p*) 5.73], and nitrogen metabolism [− log (*p*) 4.49]. The intermediates involved in the starch and sugar metabolism were highly accumulated upon laser irradiation. Besides, intermediates of carbon fixation and photosynthetic processes such as alanine and aspartate were highly up-regulated. Fatty acid biosynthesis was also found to be altered due to laser irradiation. Further, the comprehensive metabolic map was constructed and it is showing the identified primary and its associated secondary metabolites with the abundance value of laser-irradiated seeds and seedlings from 1 to 28 DAI (Fig. 7).

### Photosynthetic efficiency in response to He–Ne laser irradiation

A clear morphological variation was observed in the brinjal seedlings in response to laser irradiation (Figure S5a). Compared to the control group, laser-irradiated seedlings exhibited a higher net photosynthetic rate with a fold change of 0.84 (***p < 0.001) (Figure S5b). Significant variation in the stomatal conductance was observed in the laser-irradiated group (***p < 0.001) and the lowest level was noted in control seedlings (Figure S5c). Similarly, the transpiration rate was highest in the laser-irradiated groups (***p < 0.001) in comparison with the control (Figure S5d). A similar pattern was noted in the intercellular CO_2_ level, where the laser-irradiated seedlings showed a substantially higher amount (***p < 0.001) over the control group (Figure S5e).

## Discussion

Seed germination to seedling development is a complex process regulated by external and internal signals. The impact of laser light in the developmental processes is critical and it is necessary to understand the molecular mechanism involved in various processes. The two major phytohormones, such as gibberellic aid (GA) and abscisic acid (ABA) is known as endogenous regulators for seed germination, dormancy, plant growth and development. To analyze the impact of He–Ne laser irradiation on germination and development, these two phytohormones were quantitatively analyzed using HPLC during different developmental stages. The gradual increase in GA content and decline of ABA content was noted from germination to seedling development, and the result showed the antagonistic regulation of GA and ABA. The quantitative analysis also showed a substantial increase in the GA content in laser-irradiated groups than in un-irradiated control. The ABA is known to be the dormancy inducer, showed a significant decrease in laser groups than un-irradiated seeds. Our results were following antagonistic regulation of these two phytohormones during seed germination as reported earlier^38,39^. However, the quantitative analysis of phytohormones in plants with laser irradiation is limited. A study on the bio-stimulatory role of He–Ne laser on *Acacia farnesiana* with quantitative analysis of GA, indole acetic acid and ABA level, showed the antagonistic regulation of GA and ABA after 48 h of irradiation. The higher GA content and induced the GA_3_ β-hydrolase genes, which activate the formation of GA was observed with red laser irradiation only and not with polychromatic lights in fennel and coriander plants^40^. The gradual increase in GA and the decline of ABA content were further validated with the GA biosynthesis genes. The present results thus corroborated with the upregulation of GA synthesis genes, GA 3-oxidase (*GA3ox1* and *GA3ox2*) in laser-irradiated seeds and seedlings. The endogenous content of GA quantified through RP-HPLC was in accordance with the expression pattern of GA biosynthesis genes. Red light-induced GA levels were observed in lettuce and *Arabidopsis* by activating the transcript level of *GA3ox1* and *GA3ox2*^41,42^. The expression analysis of two ABA catabolic genes by quantitative RT-PCR revealed that *CYP707A1/A2* was photo reversibly regulated by laser irradiation and consistent with the endogenous level of ABA. The ABA level was found to be highly downregulated in the laser-irradiated seeds and seedlings, which reflects in the expression analysis of the ABA catabolic genes. Our results are corroborated with far-red/red (FR/R) light-regulated photo reversible response of ABA biosynthesis gene, *NCED6,* and the inactivation gene, *CYP707A2* in *Arabidopsis*^28^. Since ABA and GA are involved in seed germination and other developmental stages, these hormones were evaluated in seeds till the germination among control and laser-irradiated groups and subsequently in leaves during the developmental stages among control and laser-irradiated groups. Several studies revealed the differential expression of phytohormones especially GA and ABA during seed germination and growth of seedlings. ABA was found to be lower during seed germination stage and early seedling developmental stages and negatively correlated with seed filling^43^ and elongation of internodes^44^, however, ABA increases when the growth of the plant ceases^43^. GA was positively correlated with elongation during internodal development^44^. Different phytohormones including GA and ABA are responsible for the differential response of the plant to light and darkness^45^.

Plant growth, development, and reproduction are associated with several enzymes and hormones such as cytokinin and GA. The red light of laser irradiation plays an inevitable role in the synthesis and endogenous content of GA3 and GA1^46^. Further laser irradiation is also involved in increasing nitrogen level which leads to higher protein content to increase plant organs such as number of leaves and leaf area, number of branches, and umbels^47,48^. The increased leaf area, which is the reflection of laser rays enhanced cell division and elevated level of GA and subsequent growth during vegetative stage^49–51^ and improved morphological as well as physiological features^52^. The laser-mediated elevation of GA3 associated with several physiological functions such as cell elongation, auxin, and sugar content and subsequently increased the plant height, number of branches, and flowers^53,54^.

Phytochrome is an essential photoreceptor, which plays a pivotal role in red light-mediated seed germination and development. Several studies indicated the phytochrome-mediated regulation of GA and ABA metabolism, which subsequently regulates the seed germination and developmental process. The interaction of red-light absorbing photoreceptor, phytochrome in germinating seeds, plant growth, and development was reported^55^. The activation of the phytochrome system and responses was noted at a large distance in lettuce^56^ with red-light emitting He–Ne laser (632.8 nm). In the present study, the expression analysis showed that the *PhyB1* was highly upregulated in response to low-level laser irradiation. Thus, it indicated the *PhyB1* mediated regulation of seed germination in the initial hours, unlike *PhyA* or *PhyB2*. Tomato *PhyB1* strongly responds to the continuous red light, which is similar to *Arabidopsis* phytochrome B in a less dominant way^57^. Using the null mutants of phytochrome A and B in *Arabidopsis* has shown that the red-absorbing active form of *PhyB* inhibits the *PhyA*-mediated germination in response to red light treatment^58^. The influence of phytochrome B during the early stages of seed germination^33,59^ is also in accordance with our present study. The *PhyA* was highly upregulated in the irradiated seedlings from 7 to 21 DAI, indicated the *PhyA* mediated regulation on the later stages of development. The investigation of the physiological role of *PhyA* over the years showed that it regulated Very Low Fluence Responses (VLFR) and FR-HIR (High Irradiance Responses) germination. The *PhyA* can induce VLFR responses by any wavelengths from 300 to 780 nm^60^.

The early and improved growth traits of seedlings in response to laser signal perception and the higher expression of *PhyA* which induces apical hook unfolding, cotyledon opening, and activates phototropic growth^61^. The current study is the first to report the expression analysis of brinjal (Solanaceae) specific phytochrome–phytohormones regulating genes in response to low-level laser irradiation and development. The functional interaction of phytochrome with phytohormones is highly associated with various physiological processes^62^. The initial studies showed the interaction of GA signaling DELLA protein with phytochrome-interacting transcription factors (PIF) namely, PIF3 and PIF4. During red light signaling, the phytochromes interact with PIFs, whereas DELLA proteins ubiquitinated in response to GA signal and allow the PIFs to bind to the target proteins and induce germination processes^63^. In the current study, the quantification of phytochrome and phytohormones and their transcripts expression revealed the involvement of these bioactive molecules in response to He–Ne laser treatment. Hence, we hypothesize the functional interaction of these two signaling pathways for the accelerated seed germination and rapid growth of seedlings after laser irradiation, such as red light signaling for which, further studies in this line are warranted.

The influence of laser on the primary and secondary metabolites of brinjal has never been reported earlier. In the present study, the metabolite profiling was performed for the seeds and seedlings during different developmental stages, and a comparative analysis of identified metabolites was accomplished based on the abundance value in response to He–Ne laser irradiation. A total of 1237 metabolites were identified from 1 to 28 DAI of seeds and seedlings of control and irradiated groups. The amino acids and sugar metabolites were observed to be highly accumulated in seeds during the initial hours after laser irradiation. The accumulation of 9 amino acids, especially the alanine and aspartate which are synthesized by the transamination of oxaloacetate and pyruvate; shikimate derived phenylalanine, tryptophan and tyrosine were highly abundant in laser-irradiated seeds. The observation might be associated with the higher mRNA levels coding for amino acid biosynthesis which was also implicated in the study using the pathway analysis, which indicated the existence of amino acids as their free form rather than incorporated as the storage proteins. The reduction in the level of glutamate might be mainly due to its metabolic interconversion into other amino acids like glutamine and gamma-aminobutyric acid (GABA). GABA, the non-proteinogenic amino acid which elevates the nitrogen uptake and balances the carbon–nitrogen cycle, regulates plant growth and amino acids and carbohydrate metabolism^63^ was found to be upregulated in the early stages of seedling development in the laser-irradiated group.

Tryptophan accumulation evidently in the laser-irradiated group than in the control during seed to seedling transition, indicating the photosynthesis is active and it is higher in the laser-irradiated seedlings. This observation is corroborated with the physiological traits analyzed in the study in which the pigments involved in photosynthesis and the photosynthetic parameters were significantly higher in laser-irradiated seedlings. Furthermore, the drastic accumulation of nitrogen-rich amino acids (Asn, Arg), and shikimate derived amino acids (Trp, Tyr) were observed. The high abundance of amino acids from seed to seedlings was observed in the laser-irradiated group shows the amino acids are maintaining the carbon–nitrogen balance in the plant system. Further, the carbohydrate accumulation was higher in laser-irradiated seeds than the control seeds. Sucrose, raffinose, sucrose-derived glucose, and fructose showed a higher level, which indicated that the higher carbon status of the seedlings during the developmental stages. Sucrose and glucose abundance were relatively stable during the developmental stages, and it plays an important role as the imperative factor to determine the energy status of the seeds and seedlings^64^. Galactitol, which is the reduction product of galactose, showed different profiling as it was reduced in the later stages of development. Additionally, the TCA cycle intermediates such as citrate/isocitrate, malate, fumarate and succinate were significantly reduced in the initial hours after irradiation could be due to the lack of sufficient oxygen availability required for mitochondrial respiration and the ATP production^65^. The trend was different from the early stages of seedlings to the final stages in the laser-irradiated seedlings.

The abundance of fatty acids was slightly higher from 7 to 28 DAI, indicating the metabolic transition of sugars into fatty acids and their simultaneous conversion into the oil. A relatively higher and stable abundance of carbohydrates and the intermediates of TCA cycle indicate the tight regulation of glycolysis and the TCA cycle towards the final stages of seedling development. Among the identified secondary metabolites, glycoalkaloid showed a prominent variation until the maturity of seedlings. Studies have been shown that the steroidal glycoalkaloid (SGA) biosynthesis in Solanaceae plants is light sensitive^66–68^. Also, photoreceptors such as phytochrome and cryptochromes are involved in cholesterol biosynthesis by regulating mevalonate (MVA) and methylerythritol 4-phosphate (MEP) pathways^69^ leads to the synthesis of SGA precursor. Similarly, the treatment of red light has enhanced the accumulation of SGAs in potato tubers and demonstrated that it acts as the signal molecules and stimulates *phyB* receptors^70^. In our present study, the accumulation of α-solanine was relatively higher in laser-irradiated seeds than in the un-irradiated control group during the initial hours after irradiation, which indicates the involvement of red laser and the phytochrome signal transduction to increase the biosynthesis of SGA. The α-solanine accumulation was decreased tremendously in laser-irradiated leaves than control leaves during the later stage of development.

Recently, Wang et al.^71^ showed that the pigment and SGA biosynthesis pathways have common intermediates, known as isopentenyl pyrophosphate (IPP), which is imperative for the SGA biosynthesis, which occurs in the cytoplasm via the mevalonate pathway, whereas the pigment biosynthesis regulates via the MEP pathway in the chloroplasts of brinjal leaves. Compartmentalization of these processes could be the reason for the reduction of SGA accumulation in the leaves, where the pigment content is higher in the same tissues. Furthermore, the bioactive molecules mediating chlorophyll biosynthesis, geranylgeranyl chlorophyll was highly abundant from 7 to 28 DAI. The elevation in the abundance of geranylgeranyl chlorophyll is a clear indication of the higher chlorophyll synthesis in seedlings irradiated with low-level laser irradiation and it substantially validates the physiological measurement of pigment content. Further, gibberellin A12-aldehyde, an important bioactive molecule that is the precursor of GA synthesis in the plants was detected^72–74^. Thus, the endogenous content of GA in our study showed a direct relationship with the accumulated GA12-aldehyde in the tissues during different developmental stages.

The metabolic pathway analysis was evaluated to understand the significantly altered metabolites-associated pathways, and the most enriched pathways at the seed stage were aminoacyl t-RNA biosynthesis, starch and sucrose metabolism, alanine aspartate, and glutamate metabolism, TCA cycle and nitrogen metabolism. In the present study, 13 and 11 amino acids were detected which was involved in the restoration of protein synthesis in the 1 and 7 DAI respectively (asparagine, phenylalanine, glutamine, aspartic acid, valine, alanine, isoleucine/leucine, threonine, tryptophan, tyrosine, proline, glutamic acid). Seven metabolites were identified in alanine, aspartate and glutamate metabolism (aspartic acid, alanine, glutamine, glutamic acid, asparagine, fumaric acid, and succinic acid) and these metabolites were highly abundant in laser-irradiated seeds at 7 DAI. The high abundance of glutamate, which is the precursor of chlorophyll biosynthesis^75^ was noted in 7 DAI, indicate that seeds are turned to be photoautotrophic. The higher abundance of metabolites involved in the starch and sucrose metabolism (sucrose, glucose, trehalose and maltose) shows the energy required for seed germination and development. The metabolic transition observed in seed to seedling development has been reflected in the metabolite enrichment analysis. Apart from the primary metabolic pathways such as aminoacyl tRNA biosynthesis, TCA cycle and amino acid metabolism, alterations in the glucosinolate biosynthesis and phenylpropanoid pathway were observed during seedling development. Interestingly, at 21 DAI the most enriched pathway noted was phenylalanine metabolism [− log (*p*) 5.82], which is considered to be the intermediate metabolite between primary and secondary metabolism in plants^76^. The un-targeted metabolite profiling of the seeds and seedlings of brinjal in response to low-level laser irradiation demonstrated the metabolite shift occurs in an accelerative manner in laser-irradiated seeds and seedlings.

A co-relation of pigment content, photosynthesis and hormone regulation at a physiological level with altered metabolite content was observed during the later stage of development. The pigments and photosynthetic efficiency of the plant system are vital for its growth and development and therefore the photosynthetic capacity of the seedlings from all the experimental and control groups was analyzed. The total photosynthetic rate was in accordance with the pigment content in the leaves of control and irradiated groups to our earlier reports^3^. The present results conform with He–Ne laser up-regulated the photosynthesis-related genes, photosystem II PsbR protein, chlorophyll *a*/*b* binding protein, and ATP synthase^10,11^. Laser rays enhanced pigment content; photosynthetic electron transport chain efficiency was noted in wheat exposed to UV-B irradiation through enhancement of photosynthetic pigment biosynthesis capacity^10,14^. A similar observation stated that the laser irradiation might interact with the chlorophyll biosynthetic gene, mainly involved in the protochlorophyllide synthesis^33^. The current study thus substantiated the data showing the potential of laser to activate the photosystem I and II in the brinjal plants, enhancing the pigment synthesis and improved photosynthetic rate correspondingly. Further, stomatal conductance, transpiration rate and intracellular CO_2_ concentration also increased substantially in laser-irradiated groups than in the control.

## Conclusion

In conclusion, when the dormant brinjal seeds were irradiated with low-level He–Ne laser irradiation, *PhyB1* the major phytochrome accumulated in the seeds, mediated Red/far red photo reversible response to promote seed germination in the early phase. Pfr formed by red laser light increases GA levels and degrades ABA level, which was regulated through ABA catabolic and GA activating genes. The antagonistic regulation of these two phytohormones resulted in the breakage of dormancy and enhanced seed germination. The expression study further revealed the *PhyA* dependant seedling development, which resulted in the elevated photosynthetic rate. The need for primary metabolites and protein to cope with the energy requirement of the plant system for growth, photosynthesis, and development could be the reason for the variation in the primary metabolites. GA also enhances the sugar concentration of the system to boost growth and development. The study thus proved the use of the He–Ne laser as a potential tool to enhance germination and growth, which is characterized by the substantial improvement in the phytohormone, phytochrome, photosynthetic rate, and metabolites machinery.

## Methods

### Plant materials

The seeds of brinjal (*Solanum melongena L.*) var. Mattu Gulla was collected from ripe fruits and from farmers at Mattu Village, Udupi, India with prior permission and the present study is in compliance with relevant guidelines and legislation.

### He–Ne laser irradiation

The seeds were surface-sterilized for laser irradiation and irradiated with He–Ne laser (25 J/cm^2^) as a single exposure. The laser setup and the parameters used were provided in detail elsewhere^3,7^. The block diagram of the experimental setup used in the study is given in Figure S6. The seeds and seedlings were divided into different experimental groups based on the day after laser irradiation (DAI). The study groups are as follows: 0-day group in which seeds were used immediately after laser irradiation, 7th day seeds in which the germination started, followed by 14th, 21st and 28th day old seedlings (0- 28 DAI). The date of the first count was started immediately after irradiation (0 h), followed by the first, second, third, and fourth (last) count was recorded on 7, 14, 21, and 28 days respectively after irradiation. The seeds for 7–28 DAI groups were inoculated on MS^77^ basal media as described earlier^3,7^. Germination index, germination time and seed vigor index were calculated during seed germination. The 0 and 7 DAI seeds were profiled in which germinating seeds of 7 DAI with fully opened cotyledons in the laser-irradiated group. The 14–28 DAI, was the seedling stage in which the leaf was used for the metabolite characterization among the laser-irradiated and control groups.

### Extraction and quantification of hormones using HPLC

The seed (0 and 7 DAI) and leaf tissues (14, 21, and 28 DAI) from the control and irradiated group (25 J/cm^2^) were harvested. 500 mg of each sample was crushed into powder using liquid nitrogen. The 100 mg of powder was weighed, 500 µL of extraction solvent (100 µL of Con. HCl to 100 mL of 2-propanol and 50 mL of distilled water) and 1 mL dichloromethane (Merck LiChroSolv, India) was added. The samples were incubated in a rotary incubator at 100 rpm at 5 °C for 30 min, centrifuged at 14,000 rpm for 5 min at 4 °C, hormone extract was collected from the lower phase using a micropipette and lyophilized. The dry powder was thawed in 100% methanol (HPLC- grade) as 1.0 mg/mL stock^78^ and stored at − 20 °C till future use. The sample preparations and HPLC conditions are given in the Supplementary Information.

### RNA extraction and quantitative real-time PCR analysis

RNA was extracted from germinating seeds and seedlings after laser irradiation using Trizol reagent (Thermo Fisher Scientific, USA) according to the manufacturer’s instructions. The detailed protocol and the list of primer sequences used are described in the Supplementary file and Supplementary Table 3 respectively.

### Sample preparation and liquid chromatography coupled to mass spectrometry analysis

The seed and seedling tissues were grounded into powder using liquid nitrogen in a pre-chilled mortar and pestle. The metabolite extraction from seeds and seedlings was performed^79^. The 100 mg of powder was transferred to a pre-chilled centrifuge tube and 1 mL of ice-cold extraction solvent (99.875% methanol acidified with 0.125% FA) was added and vortex for 10 s. Samples were sonicated for 15 min at room temperature and centrifuged for 10 min at 13,000 rpm. The supernatant was filtered through a 0.2 mm PTFE filter and stored at – 20 °C for mass spectrometry analysis. The mass spectrometry conditions and analysis are given in the Supplementary Information.

### Data processing and analysis

LC–MS data from positive and negative ESI mode was aligned and normalized using Agilent G3835AA Mass Hunter Mass Profiler Professional Software version MPP 12.6.1 (Agilent Technologies, Santa Clara, California, United States). The parameters used are described in the Supplementary Information.

### Photosynthetic parameters

The young, vigorous and developed leaves (60 days old) were selected from seedlings of each experimental group for the measurement of photosynthetic rate. The photosynthesis rate was measured using a standard leaf chamber equipped with a 6400-02B LED light source in an LI-6400 portable photosynthesis system (Li-Cor, Inc. Lincoln, NE, U.S.A). Besides, the stomatal conductance, transpiration rate and intracellular CO_2_ were also measured using the Li-Cor system^80^. The calibration used for the Li-Cor analysis is given in the Supplementary Information.

### Statistical analysis

The laser-irradiated and control groups consisted of 25 seeds and the experiments were performed in triplicate. The samples used for germination assay, and photosynthetic parameters were biological replicates, and the samples for HPLC, qRT-PCR, and Mass spectrometry were technical repeats. The mean values of different parameters from laser-irradiated and control groups were imperiled to one-way ANOVA with Bonferroni’s test for multiple comparisons, and the significance between the groups was calculated. The statistical analysis was performed using biostatistics software Graph PAD Prism 5.

## Supplementary Information

Supplementary Information.

## References

[1] Qi Z, Yue M, Wang XL (2000). Laser pretreatment protects cells of broad bean from UV-B radiation damage. J. Photochem. Photobiol. B..

[2] Krawiec M, Dziwulska-Hunek A, Palonka S, Kaplan M, Baryla P (2016). Effect of laser irradiation on seed germination and root yield of scorzonera (*Scorzonera hispanica* L.). Acta Agrophys..

[3] Muthusamy A (2012). Influence of helium–neon laser irradiation on seed germination in vitro and physico-biochemical characters in seedlings of Brinjal (*Solanum melongena* L.) var. Mattu Gulla. Photochem. Photobiol. B.

[4] AlSalhi MS, Tashish W, Al-Osaif SS, Atif M (2018). Effects of He–Ne laser and argon laser irradiation on growth, germination, and physico-biochemical characteristics of wheat seeds (*Triticum aestivum* L.). Laser Phys..

[5] Jamil Y (2013). He–Ne laser-induced changes in germination, thermodynamic parameters, internal energy, enzyme activities and physiological attributes of wheat during germination and early growth. Laser Phys. Lett..

[6] Thorat SA (2021). Red laser-mediated alterations in seed germination, growth, pigments and withanolide content of Ashwagandha [*Withania somnifera* (L.) Dunal]. J. Photochem. Photobiol. B.

[7] Swathy SP (2016). Responses of He–Ne laser irradiation on agronomical characters and chlorogenic acid content of brinjal (*Solanum melongena* L.) var. Mattu Gulla. J. Photochem. Photobiol. B.

[8] Li YF, Gao LM, Han R (2017). He–Ne laser illumination ameliorates photochemical impairment in ultraviolet-B stressed-wheat seedlings *via* detoxifying ROS cytotoxicity. Russ. J. Plant Physiol..

[9] Swathy PS, Rupal G, Vijendra P, Mahato KK, Muthusamy A (2017). In vitro culture responses, callus growth and organogenetic potential of brinjal (*Solanum melongena* L.) to He–Ne laser irradiation. J. Photochem. Photobiol. B Biol..

[10] Qiu Z, Yuan M, He Y, Li Y, Zhang L (2017). Physiological and transcriptome analysis of He–Ne laser pre-treated wheat seedlings in response to drought stress. Sci. Rep..

[11] Gao L, Li Y, Shen Z, Han R (2018). Responses of He–Ne laser on agronomic traits and the crosstalk between UVR8 signaling and phytochrome B signaling pathway in *Arabidopsis thaliana* subjected to supplementary ultraviolet-B (UV-B) stress. Protoplasma.

[12] Qiu ZB, Liu X, Tian XJ, Yue M (2008). Effects of CO_2_ laser pretreatment on drought stress resistance in wheat. J. Photochem. Photobiol..

[13] Chen YP (2009). Response of antioxidant defense system to laser radiation apical meristem of *Isatis indigotica* seedlings exposed to UV-B. Plant Signal. Behav..

[14] Chen H, Han R (2014). He–Ne laser treatment improves the photosynthetic efficiency of wheat exposed to enhanced UV-B radiation. Laser Phys..

[15] Liu F, Chen H, Han R (2015). The effects of He–Ne laser and enhanced ultraviolet-B radiation on proliferating-cell nuclear antigen in wheat seedlings. Am. J. Plant Sci..

[16] Li Y, Gao L, Han R (2016). A combination of He–Ne laser irradiation and exogenous NO application efficiently protect wheat seedling from oxidative stress caused by elevated UV-B stress. Environ. Sci. Pollut. Res..

[17] Gao LM, Li YF, Han R (2015). He–Ne laser preillumination improves the resistance of tall fescue (*Festuca arundinacea* Schreb.) seedlings to high saline conditions. Protoplasma.

[18] Li Y, Gao L, Han R (2016). Endogenous nitric oxide mediates He–Ne laser-induced adaptive responses in salt stressed-tall fescue leaves. Biosci. Biotechnol. Biochem..

[19] Truchliński J, Wesolowski M, Koper R, Dziamba S (2002). Influence of pre-sowing red light radiation on the content of antinutritional factors, mineral elements and basic nutritional component contents in triticale seeds. Int. Agrophys..

[20] Abbas M (2017). Muscilage characterization, biochemical and enzymatic activities of laser irradiated *Lagenaria siceraria* seedlings. J. Photochem. Photobiol. B.

[21] Perveen R (2010). Effects of different doses of low power continuous wave He–Ne laser radiation on some seed thermodynamic and germination parameters, and potential enzymes involved in seed germination of sunflower (*Helianthus annuus* L.). Photochem. Photobiol..

[22] Perveen R (2011). He–Ne laser-induced improvement in biochemical, physiological, growth and yield characteristics in sunflower (*Helianthus annuus* L.). Photochem. Photobiol..

[23] Kumar G, Srivastava P, Pandey JK, Gopal R (2010). Effect of laser-irradiation on photosynthetic efficiency of safflower leaves. J. Phytol..

[24] Gao, B. & Zhang, C. A preliminary physiological and biochemical study on He–Ne laser mutation breeding of *Erigeron breviscapus*. In *Seventh International Conference on Photonics and Imaging in Biology and Medicine*, Vol. **7280,** 72802 (International Society for Optics and Photonics, 2009). https://doi.org/10.1117/12.821431.

[25] Rybinski W, Garczynski S (2004). Influence of laser light on leaf area and parameters of photosynthetic activity in DH lines of spring barley (*Hordeum vulgare* L.). Int. Agrophys..

[26] Shaban N, Kartalov P (1988). Effect of laser irradiation of seeds on some physiological processes in cucumbers. Rast. Nauk..

[27] Cholakov D, Uzunov N, Meranzova R (1997). The effect of the helium–neon laser irradiation of cucumber seeds on the physiological characters of plants grown under water stress conditions. I Balk. Symp. Veg. Potatoes.

[28] Seo M (2006). Regulation of hormone metabolism in *Arabidopsis* seeds: phytochrome regulation of abscisic acid metabolism and abscisic acid regulation of gibberellin metabolism. Plant J..

[29] Tepperman JM, Hudson ME, Khanna R, Zhu T, Chang SH, Wang X, Quail PH (2004). Expression profiling of phyB mutant demonstrates substantial contribution of other phytochromes to red-light-regulated gene expression during seedling de-etiolation. Plant J..

[30] Pierik R, De Wit M (2013). Shade avoidance: phytochrome signaling and other aboveground neighbour detection cues. J. Exp. Bot..

[31] Devlin PF, Robson PR, Patel SR, Goosey L, Sharrock RA, Whitelam GC (1999). Phytochrome D acts in the shade-avoidance syndrome in Arabidopsis by controlling elongation growth and flowering time. Plant Physiol..

[32] Alba R, Kelmenson PM, Cordonnier-Pratt MM, Pratt LH (2000). The phytochrome gene family in tomato and the rapid differential evolution of this family in angiosperms. Mol. Biol. Evol..

[33] Appenroth KJ, Lenk G, Goldau L, Sharma R (2006). Tomato seed germination: regulation of different response modes by phytochrome B2 and phytochrome A. Plant Cell Environ..

[34] Särkinen T, Bohs L, Olmstead RG, Knapp S (2013). A phylogenetic framework for evolutionary study of the nightshades (Solanaceae): a dated 1000-tip tree. BMC Evol. Biol..

[35] Kim S (2014). Genome sequence of the hot pepper provides insights into the evolution of pungency in Capsicum species. Nat. Genet..

[36] Barchi L, Pietrella M, Venturini L (2019). A chromosome-anchored eggplant genome sequence reveals key events in Solanaceae evolution. Sci. Rep..

[37] Finch-Savage WE, Leubner-Metzger G (2006). Seed dormancy and the control of germination. New Phytol..

[38] Ouf SA, Abdel-Hady NF (1999). Influence of He−Ne laser irradiation of soybean seeds on seed mycoflora, growth, nodulation, and resistance to *Fusarium solani*. Folia Microbiol..

[39] Liu X, Hou X (2018). Antagonistic regulation of ABA and GA in metabolism and signaling pathways. Front. Plant Sci..

[40] Shu K, Liu XD, Xie Q, He ZH (2016). Two faces of one seed: hormonal regulation of dormancy and germination. Mol. Plant.

[41] El Tobgy KM, Osman YA, El Sherbini EA (2009). Effect of laser radiation on growth, yield and chemical constituents of anise and cumin plants. J. Appl. Sci. Res..

[42] García-Martinez JL, Gil J (2001). Light regulation of gibberellin biosynthesis and mode of action. J. Plant Growth Regul..

[43] Yan B, Hou J, Cui J, He C, Li W, Chen X, Li M, Wang W (2019). The effects of endogenous hormones on the flowering and fruiting of *Glycyrrhiza uralensis*. Plants.

[44] Patel D, Thaker VS (2007). Estimation of endogenous contents of phytohormones during internode development in *Merremia emarginata*. Biol. Plant..

[45] Golovatskaya IF, Karnachuk RA (2007). Dynamics of growth and the content of endogenous phytohormones during kidney bean scoto-and photomorphogenesis. Russ. J. Plant Physiol..

[46] Kamiya YLJ, Martinez G (1999). Regulation of gibberellin biosynthesis by light. Curr. Opin. Plant Biol..

[47] Osman YAH, EL-Tobgy KMK, El-Sherbini EA (2009). Effect of laser radiation treatments on growth, yield and chemical constituents of fennel and coriander plants. J. Appl. Sci. Res..

[48] Noha SK, El Ghandoor H (2011). Investigate the effect of Nd-Yag laserbeam on soybean (*Glycin max*) leaves at the protein level. Int. J. Biol..

[49] Rybiñski W, Garczyñski S (2004). Influence of laser light on leaf area and parameters of photosynthetic activity in DH lines of spring barley (*Hordeum vulgare* L.). Int. Agrophys..

[50] El-Kereti MA, El-feky SA, Khater MS, Osman YAH, El-Sherbini EA (2013). ZnO nanofertilizer and He–Ne laser irradiation for promoting growth and yield of sweet basil plant. Recent Pat. Food Nutr. Agric..

[51] Al-sherbini A, Abd-El-Gawad HG, Kamal MA, Souad AEF (2015). Potential of He–Ne laser irradiation and iron nanoparticles to increase growth and yield of pea. Agric. Environ. Sci..

[52] Możdżeń K, Barabasz-Krasny B, Zandi P (2020). Effect of long-term of He–Ne laser light irradiation on selected physiological processes of Triticale. Plants.

[53] Lobna ST, Hanan AAT, Metwally SA, Hwida MF (2014). Effect of laser radiation treatments on in vitro growth behavior, antioxidant activity and chemical constituents of *Sequoia sempervirens*. Res. J. Pharm. Biol. Chem. Sci..

[54] Rania AT, Lobna ST, Metwally SA (2015). In vitro cultures of jojoba (*Simmondsia chinensis* L.) affecting by laser irradiation. J. Chem. Biol. Phys. Sci..

[55] Yamaguchi S, Kamiya Y (2001). Gibberellins and light-stimulated seed germination. J. Plant Growth Regul..

[56] Seo M, Nambara E, Choi G, Yamaguchi S (2009). Interaction of light and hormone signals in germinating seeds. Plant Mol. Biol..

[57] Paleg LG, Aspinall D (1970). Field control of plant growth and development through the laser activation of phytochrome. Nature.

[58] Weller JL, Schreuder ME, Smith H, Koornneef M, Kendrick RE (2000). Physiological interactions of phytochromes A, B1 and B2 in the control of development in tomato. Plant J..

[59] Shinomura T, Nagatani A, Chory J, Furuya M (1994). The induction of seed germination in *Arabidopsis thaliana* is regulated principally by phytochrome B and secondarily by phytochrome A. Plant Physiol..

[60] Lee KP (2012). Spatially and genetically distinct control of seed germination by phytochromes A and B. Genes Dev..

[61] Casal JJ, Candia AN, Sellaro R (2013). Light perception and signalling by phytochrome A. J. Exp. Bot..

[62] Sheerin DJ, Hiltbrunner A (2017). Molecular mechanisms and ecological function of far-red light signalling. Plant Cell Environ..

[63] Neff, M.M., Street, I.H., Turk, E.M. & Ward, J.M. Interaction of light and hormone signalling to mediate photomorphogenesis. In *Photomorphogenesis in Plants and Bacteria*, 439–473 (Springer, Dordrecht, 2006). https://doi.org/10.1007/1-4020-3811-9_21.

[64] De Lucas M (2008). A molecular framework for light and gibberellin control of cell elongation. Nature.

[65] Cheng B (2018). The γ-Aminobutyric acid (GABA) alleviates salt stress damage during seeds germination of white clover associated with Na^+^/K^+^ transportation, dehydrins accumulation, and stress-related genes expression in white clover. Int. J. Mol. Sci..

[66] Quéro A (2016). Metabolite profiling of developing *Camelina sativa* seeds. Metabolomics.

[67] Fait A (2006). Arabidopsis seed development and germination is associated with temporally distinct metabolic switches. Plant Physiol..

[68] Percival GC (1999). The influence of light upon glycoalkaloid and chlorophyll accumulation in potato tubers (*Solanum tuberosum* L.). Plant Sci..

[69] Zrust J, Horackova V, Prichystalova V, Rejlkova M (2001). Light-induced alpha-chaconine and alpha-accumulation in potato tubers (*Solanum tuberosum*) after harvest. Rostlinna Vyroba-UZPI (Czech Republic).

[70] Machado RM, Toledo MCF, Garcia LC (2007). Effect of light and temperature on the formation of glycoalkaloids in potato tubers. Food Control.

[71] Lichtenthaler HK (1999). The 1-deoxy-D-xylulose-5-phosphate pathway of isoprenoid biosynthesis in plants. Ann. Rev. Plant Biol..

[72] Wang CC, Sulli M, Fu DQ (2017). The role of phytochromes in regulating biosynthesis of sterol glycoalkaloid in eggplant leaves. PLoS ONE.

[73] Kobayashi M, Spray CR, Phinney BO, Gaskin P, MacMillan J (1996). Gibberellin metabolism in maize (the stepwise conversion of gibberellin a12-aldehyde to gibberellin a20. Plant Physiol..

[74] Van Den Berg JH, Davies PJ, Ewing EE, Halinska A (1995). Metabolism of gibberellin A12 and A12-aldehyde and the identification of endogenous gibberellins in potato (*Solanum tuberosum* ssp. andigena) shoots. J. Plant Physiol..

[75] Reinbothe S, Reinbothe C (1996). The regulation of enzymes involved in chlorophyll biosynthesis. Eur. J. Biochem..

[76] Tzin V, Galili G (2010). The biosynthetic pathways for shikimate and aromatic amino acids in *Arabidopsis thaliana*. Arabidopsis Book.

[77] Murashige T, Skoog F (1962). A revised medium for rapid growth and bio assays with tobacco tissue cultures. Physiol. Plant..

[78] Pan X, Welti R, Wang X (2010). Quantitative analysis of major plant hormones in crude plant extracts by high-performance liquid chromatography–mass spectrometry. Nat. Protoc..

[79] De Vos RC (2007). Untargeted large-scale plant metabolomics using liquid chromatography coupled to mass spectrometry. Nat. Prot..

[80] Evans JR, Santiago LS (2014). PrometheusWiki Gold Leaf Protocol: gas exchange using LI-COR 6400. Funct. Plant Biol..

