# Supplementary material for: He–Ne laser accelerates seed germination by modulating growth hormones and reprogramming metabolism in brinjal
Source: Sci Rep. 2021 Apr 12;11:7948. doi: 10.1038/s41598-021-86984-8 (PMC8042036; doi:10.1038/s41598-021-86984-8)
Supplement: Supplementary file 1 — Supplementary Information. [file 41598_2021_86984_MOESM1_ESM.pdf]

He-Ne laser accelerates seed germination by modulating growth hormones and reprogramming metabolism in brinjal

Puthanvila Surendrababu Swathy[1], Kodsara Ramachandra Kiran[1], Manjunath B Joshi[2], Krishna Kishore Mahato[3], Annamalai Muthusamy[1*]

[1]Department of Plant Sciences, Manipal School of Life Sciences, Manipal Academy of Higher Education, Manipal -576104, Karnataka, India.

[2]Department of Ageing Research, Manipal School of Life Sciences, Manipal Academy of Higher Education, Manipal -576104, Karnataka, India.

[3]Department of Biophysics, Manipal School of Life Sciences, Manipal Academy of Higher Education, Manipal -576104, Karnataka, India.

Materials and methods:

Plant materials and Laser Irradiation. The seeds and seedlings were divided into different experimental groups based on the day after laser irradiation (DAI). The study groups are as follows: 0-day group in which seeds were used immediately after laser irradiation, 7th-day seeds in which the germination started, followed by 14th, 21st and 28th day (0- 28 DAI). The seeds for 7-28 DAI groups were inoculated on MS[78] basal media as described earlier[7]. Germination index, germination time and seed vigour index were calculated during seed germination.

Photosynthetic Parameters. The LI-6400 portable photosynthesis system was calibrated for flow rate, pressure, temperature and humidity before the readings. The leaf was placed in the chamber/cuvette with an air concentration of 21 % O_2, 400 μmol mol^{-1} CO_2, 60% relative moisture and 30 °C [81].

Extraction and quantification of Hormones using HPLC.

HPLC was performed using a Waters 2695 separating module system (Waters India Pvt Ltd). The separations were carried out on a 250 mm×4.6 mm C18 column with the column and sample temperature was maintained at 20 °C and 4 °C respectively. The isocratic conditions were from 0-20 min, 65% of solvent A and 35% solvent B for gibberellic acid, and from 0-10 min, 40% of solvent A and 60% solvent B for abscisic acid. The flow rate was adjusted to 1 mL/min with 20 μL of samples was injected in triplicate. The samples were detected using Waters 2487 dual-wavelength absorbance UV detector at a wavelength of 206 nm for gibberellic acid and 254 nm for abscisic acid. The quantification was calculated based on the area under the peak and the data analysis was performed using Empower 2 pro software.

RNA extraction and quantitative real-time PCR analysis.

The RNA was quantified using nanodrop spectrophotometry (Thermo Fisher Scientific, USA) and stored at -80 °C till further use. Prior to the cDNA conversion, 2 µg of RNA was treated with 2 U DNase I (RNase-free) (Invitrogen) as per the manufacturer's protocol. The treated RNA samples were reverse transcribed to cDNA using high capacity cDNA transcription kit (Applied Biosystems Inc., Foster City, CA) and stored at -80 ºC until further use. Quantitative Real-time PCR was performed using the KAPA SYBR FAST qPCR kit on the 7500 Fast real-time PCR system (Applied Biosystems). The relative transcript level of GA (*GA3ox1* and *GA3ox2*), ABA (*CYP707A1* and *CYP707A2*) and phytochrome (*PhyA*, *PhyB1* and *PhyB2*) was calculated by the comparative ΔCt and normalized to 18s rRNA transcript levels. The primers were designed by Primer 3 Input (version 0.4.0) and the primer sequences are listed in Supplementary Table 1.

Sample Preparation and Liquid Chromatography coupled to Mass Spectrometry analysis. Un targeted small molecule profiling was carried out in Agilent 6530 Accurate-Mass Q-TOF (Agilent Technologies, Santa Clara, California, United States) coupled with a high-performance liquid chromatography system (Agilent 1200 series, Santa Clara, USA). The separation was accomplished on a 250 mm × 4.6 mm C18 column (Phenomenex-Luna) with 0.1% formic acid (FA) in ultrapure water (v/v) (solvent A) and 0.1% FA in acetonitrile (Solvent B) as mobile phases. The flow rate was 0.5 mL/min and the run was for 70 min with the injection volume of 5 µL. The gradient elution was carried out using: 0–45 min, 5% solvent B; 45–47 min, 5–35% B; 47–52 min, 75% B; 52-54 min, 75-5% B and 54-70 min, 5% B. Electrospray ionization (ESI) was used as ion source with the following parameters: Capillary voltage 3000 V, dry gas temperature 350 °C and dry gas flow rate 8 L/min. The profiling was performed in both positive and negative ESI mode in duplicates. The ion range was given

within a range of 60 to 1500 mass to charge ratio (m/z) by the Mass Hunter Workstation Software version B.04.00 (Agilent Technologies, Santa Clara, California, United States).

The metabolic pathway analysis

The pathway analysis was carried out using Metaboanalyst (MetPA) with the identified metabolites from each developmental stage to identify the most relevant metabolic pathways involved in response to laser irradiation. The compound name was provided for the analysis with *Arabidopsis thaliana* as a pathway library. Overrepresentation analysis was performed using the hypergeometric test to avoid the repetition of compounds provided. The pathway topology analysis was performed to estimate the node importance using Relative-betweenness centrality.

Data Processing and Analysis

The parameters used were as follows: minimum absolute abundance as 5000 counts, a minimum number of ions was 2, retention time window as 0.5 min and the mass window was 2 mDa. The normalized data were subjected to Principal Component Analysis (PCA) and orthogonal partial least squares-discriminant analysis (OPLS-DA) using Metaboanalyst software. Further, the OPLS-DA tool used for dimension reduction and identification of spectral features for group separation was applied. The metabolites present at least in 50% of the samples from each experimental group were subjected to metabolite identification via plant metabolic network database (PMN 12.0) in addition to pre-published data, and other databases (METLIN). The abundance values of metabolites from control and irradiated groups were transformed into log10 values and subjected to statistical analysis an unpaired t-test (Graphpad Prism 5.0) and represented. Pathway

enrichment analysis and correlation analysis of the identified metabolites was performed using Metabolomics Pathway Analysis (MetPA) using MetaboAnalyst software 3.0.

Results

Altered germination traits in response to laser irradiation

The germination curve, index, mean germination time and seed vigour index were noted from the control and laser-irradiated groups. A significant elevation was observed in the germination curve and index of the laser-irradiated group over control (**p<0.01) (Figure S1a,b). Mean germination time was significantly reduced in the laser-irradiated groups (**p<0.01) (Figure S1c). The lower germination time indicates the rapid seed germination and seed vigour index, showed the superior seed germination, as well as rapid seedling growth, was highest for laser-irradiated groups as compared to un-irradiated control (***p<0.001) (Figure S1d).

Identification and quantification of primary metabolites

The organic acids and the intermediates of TCA cycles such as succinate, fumarate, citrate/isocitrate, and malate were exhibited a significant low abundance in the laser-irradiated seeds after 24 h incubation (Figure S2a), whereas the abundance level of these metabolites elevated significantly during the later stage of seedling development (Figure S2b-e). The most predominant fatty acids in the brinjal seed, linoleic acid was reported in the study throughout the developmental stages. During the initial days of germination (1 DAI), the level was lower in the laser-irradiated seeds, which was gradually increased by 7 DAI (Figure S2b), and linoleic acid was not detected in the leaves of the seedlings. Besides, the essential fatty acids such as stearic acid and arachidic acids showed an elevated level from the initial stages to

later stages of seedling development. The stearic acid was detected in 7 and 28 DAI, and shown an up-regulation. A higher abundance of arachidic acid was detected in the 14 and 21 DAI laser-irradiated group (Figure S2c and d).

The metabolic pathway analysis

In contrast, the intermediates of TCA cycles were down-regulated in the laser-irradiated seeds (Figure S3). The pathway analysis of identified metabolites from 7 DAI has also altered 21 pathways, which is similar to 1 DAI. In contrast to the previous phase of development, the intermediates of the TCA cycle were highly up-regulated at 7 DAI groups and the intermediates in starch and sucrose metabolism were constantly up-regulated.

The flavonoid biosynthesis pathway has also been found to be up-regulated due to laser irradiation (Figure S3b). The biochemical pathway profiling of the 14 DAI group showed the alterations in 15 pathways such as aminoacyl t-RNA biosynthesis [-log (p) 1.50], valine, leucine and isoleucine biosynthesis [-log (p) 6.39], glucosinolate biosynthesis [-log (p) 5.45], arginine and proline metabolism [-log (p) 4.97], glycine, serine and threonine metabolism [-log (p) 4.78] (Figure S5c). The alteration in 11 metabolic networks was observed at 21 DAI experimental groups with phenylalanine metabolism which found to be the most predominant pathway [-log (p) 5.82], followed by amino acyl-tRNA biosynthesis [-log (p) 5.40]. In addition, nitrogen metabolism [-log (p) 4.54], glucosinolate biosynthesis [-log (p) 4.00], galactose metabolism [-log (p) 4.48], and phenylpropanoid biosynthesis [-log (p) 2.49] was marked to be altered (Figure S3d). A total of 13 pathways showed alterations in 28 DAI groups in which the most hits of metabolites were observed in amino acyl-tRNA biosynthesis [-log (p) 6.82], galactose metabolism [-log (p) 5.63], starch and sucrose metabolism [-log (p) 5.10], and citrate cycle [-log (p) 2.40] (Figure S3e). The flavonoid biosynthesis intermediates were found to be altered with higher accumulation in the 28 DAI groups. Besides, the stilbene, diarylheptanoid and gingerol biosynthesis, and carbon fixation in the photosynthetic biosynthesis pathway were also highly up-

regulated in the laser-irradiated seedlings. Further, the comprehensive metabolic map was constructed and it is showing the identified primary and its associated secondary metabolites with the abundance value of laser-irradiated seeds and seedlings from 1-28 DAI (Figure S4a-e).

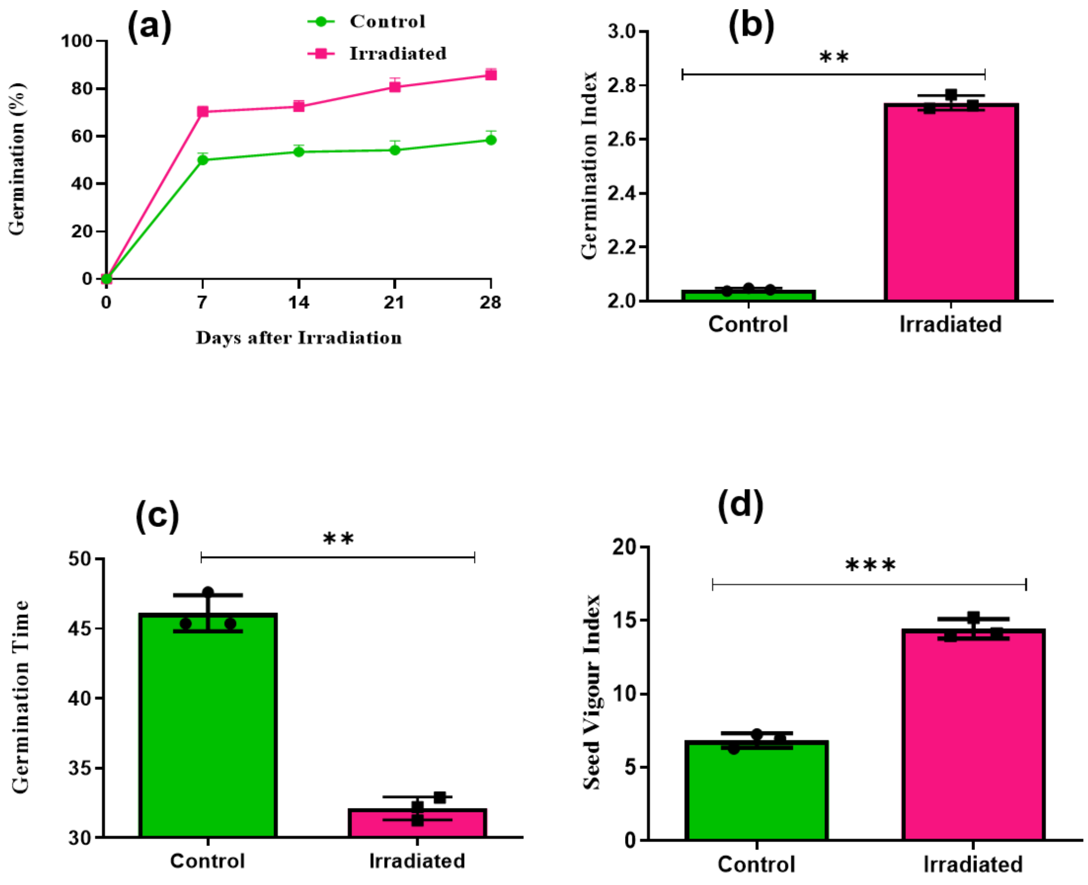

Figure S1. Germination parameters a) Germination percentage b) germination index c) germination time and d) seed vigour index of brinjal (*Solanum melongena* L.) var. Mattu Gulla in response to He-Ne laser irradiation and un-irradiated control. Data are expressed as mean ± SD and significant at *** p< 0.001 and ** p< 0.01 compared with non-irradiated control (n=3).

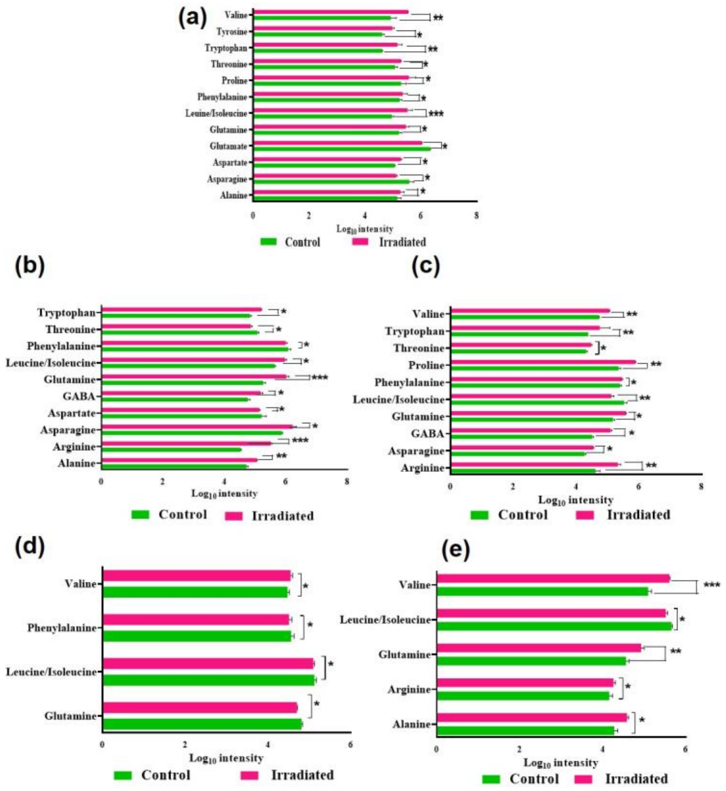

Figure S2. Metabolite intensities of most abundant and significantly altered amino acids a) 1 DAI b) 7 DAI c) 14 DAI d) 21 DAI and e) 28 DAI of seeds and seedlings of brinjal (*Solanum melongena* L.) var. Mattu Gulla in response to He-Ne laser irradiation and un-irradiated control. The values were log10 transformed. Statistically significant changes in metabolite intensity between the control and laser-irradiated groups are represented as ***p<0.001, **p<0.01, and *p<0.05 (n=2).

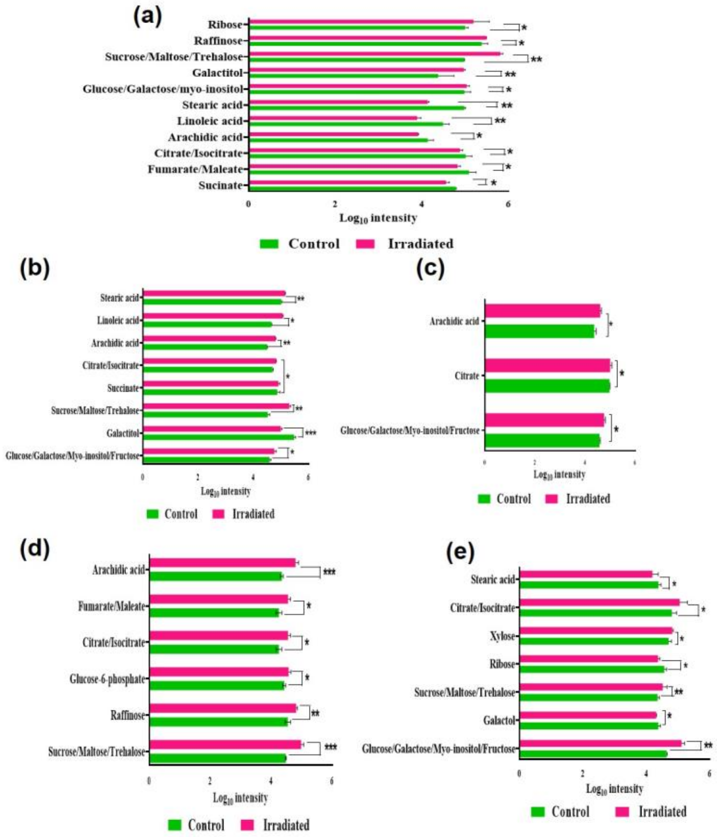

Figure S3. Metabolite intensities of most abundant and significantly altered sugar, fatty acids and organic acids a) 1 DAI b) 7 DAI c) 14 DAI d) 21 DAI and e) 28 DAI of seeds and seedlings of brinjal (*Solanum melongena* L.) var. Mattu Gulla in response to He-Ne laser irradiation and un-irradiated control. The values were log10 transformed. Statistically significant changes in metabolite intensity between the control and laser-irradiated groups are represented as ***p<0.001, **p<0.01, and *p<0.05 (n=2).

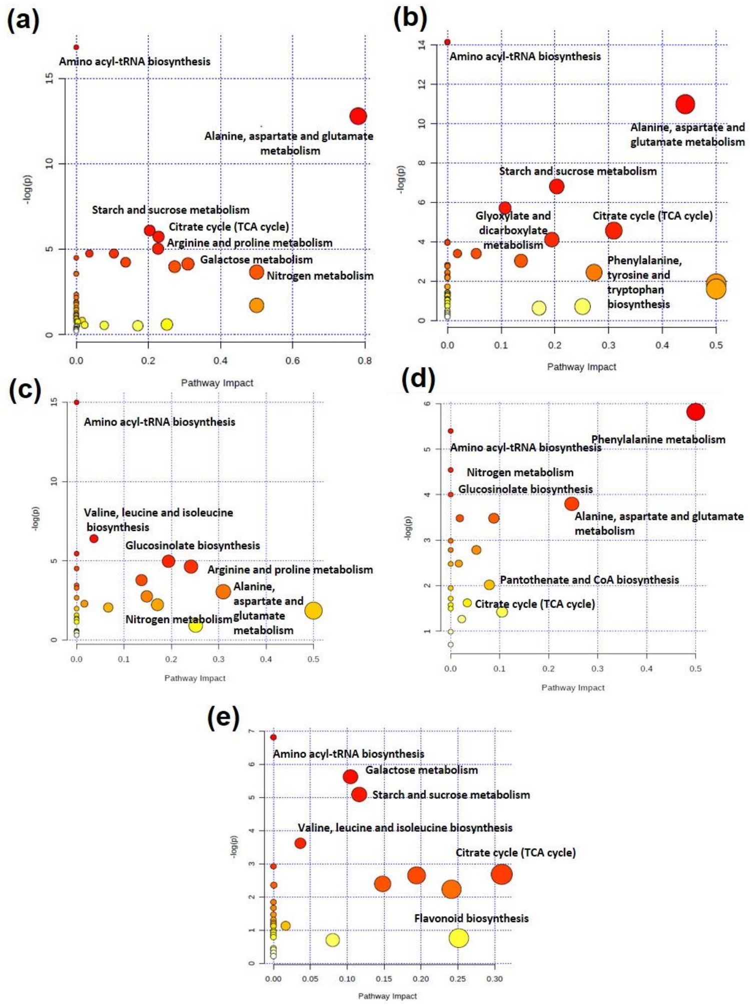

Figure S4. MetPA analysis showing pathway enrichment of metabolites identified a) 1 DAI b) 7 DAI c) 14 DAI d) 21 DAI and e) 28 DAI of seeds and seedlings of brinjal (*Solanum melongena* L.) var. Mattu Gulla in response to He-Ne laser irradiation and un-irradiated control. The size and colour of circles represent p values and pathway impact respectively.

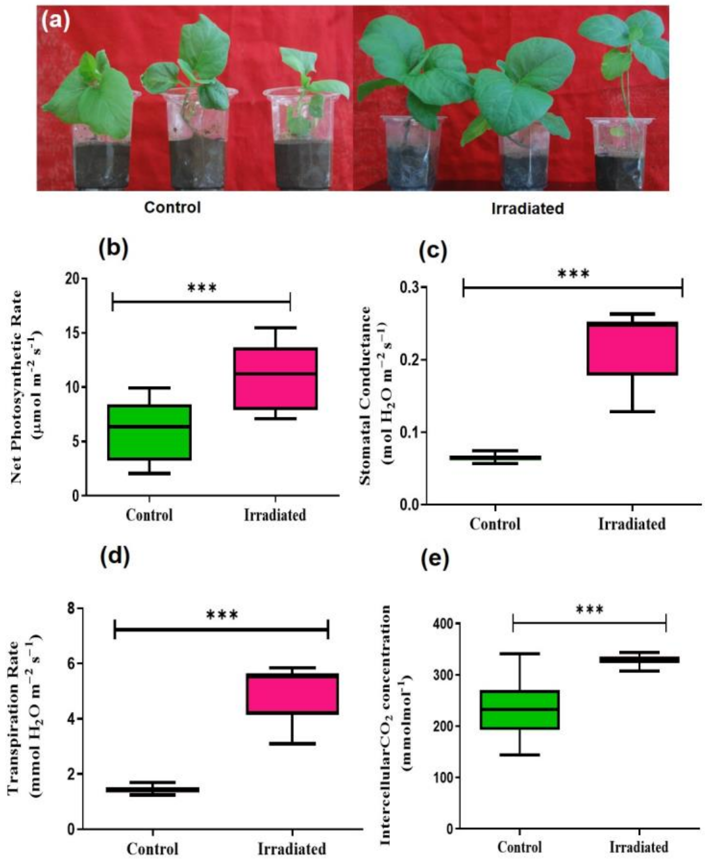

Figure S5. Photosynthetic parameters of control and laser-irradiated seedlings. a) Sixty-day old plantlets, b) Net photosynthetic rate (Pn), c) Stomatal conductance (gs), d) Transpiration rate (T) and e) Intercellular CO_2 concentration (Ci) of brinjal (*Solanum melongena* L.) var. Mattu Gulla in response to He-Ne laser and un-irradiated control (n=3). Data are expressed as mean ± SD and significant at ***$p < 0.001$, **$p < 0.01$, and *$p < 0.05$ compared with un- irradiated control.

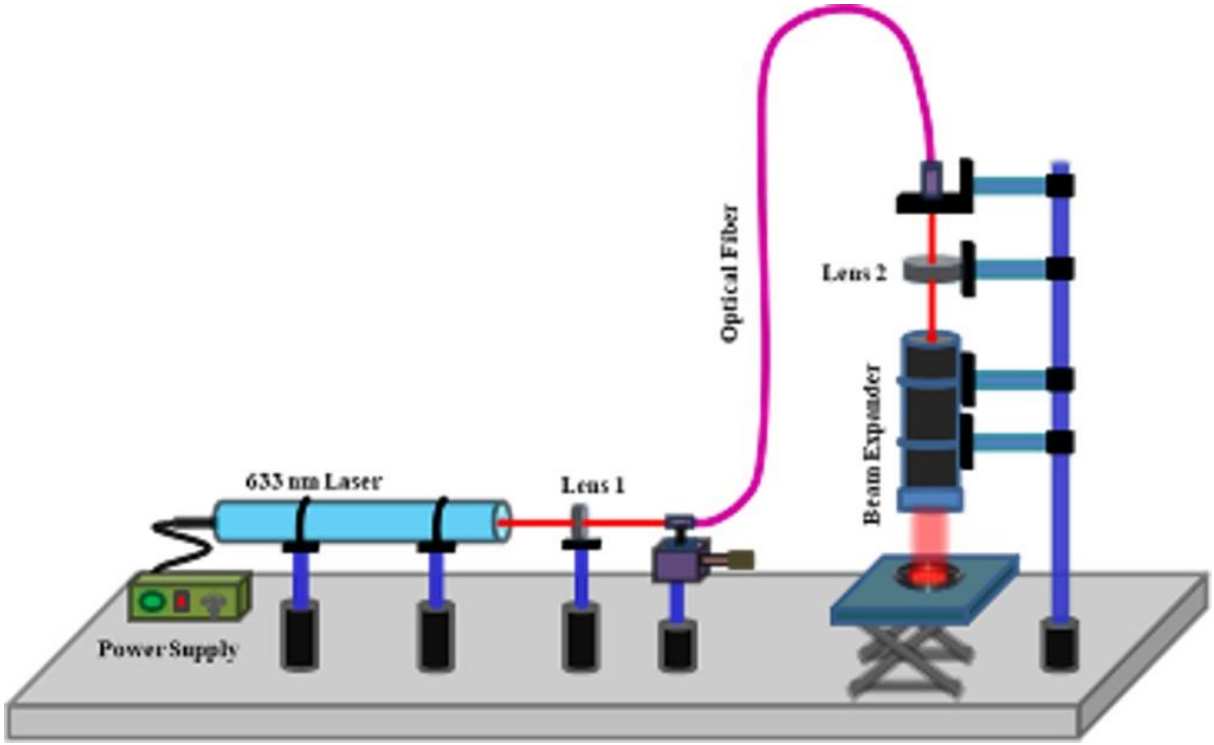

Figure S6. Diagrammatic representation of He-Ne laser source for seed irradiation.

Supplementary Table 1. Miscellaneous secondary metabolites obtained from positive and negative mode ESI in LC-MS analysis from seeds and seedlings of brinjal (*Solanum melongena* L.) var. Mattu Gulla (1-28 DAI) in control and laser-irradiated group.

1 DAI

Sl. No.	Monoisotopic Mass	Retention Time (min)	Theoretical molecular weight	ppm error	Polarity	Compound Name	Chemical Formula	Classification	Fold change	Regulation	p value
1	72.08132433	7.20	71.073499293	7.607160	Positive	Pyrrilodine	$C_4H_{10}N_1$	Amine	-0.073	DOWN	***
2	164.04734412	6.087	164.047344	0.000731	Positive	P-Coumaric Acid	$C_9H_7O_3$	Phenolic acid	-0.053	DOWN	*
3	191.0582432	16.99	191.058243159	0.000215	Negative	2-Oxo-Indole-3-Acetate	$C_{10}H_8NO_3$	Indole derivative	-0.172	DOWN	**
4	198.0892089	6.90	198.089209	-0.000505	Positive	7-Deoxyloganetate	$C_{10}H_{13}O_4$	Terpenoid	0.067	UP	**
5	221.1276601	56.10	221.12766	0.000452	Negative	Dihydrozeatin	$C_{10}H_{15}N_5O_1$	Hormone	-0.034	DOWN	***
6	251.1018393	63.31	251.101839307	-0.000028	Negative	3'-Deoxyadenosine	$C_{10}H_{13}N_5O_3$	Nucleic acid	0.655	UP	**
7	266.1518092	5.12		2.059731	Positive	(2S,5R)-2-(2-Hydroxypropan-2-Yl)-5,9-Dimethyl-1-Oxaspiro [5.5] Undec-8-Ene-7,10-Dione	$C_{15}H_{22}O_4$	Terpenoid	-0.062	DOWN	*
8	360.14024	7.39	360.157288	-47.334874	Positive	(-)-Lariciresinol	$C_{20}H_{24}O_6$	Lignan	-0.154	DOWN	***
9	496.32701	56.72	496.34000	-26.171576	Positive	26-Hydroxybrassinolide	$C_{28}H_{48}O_7$	Hormone	-0.089	DOWN	***
10	652.2003351	6.15	652.2003351092	-0.000014	Positive	Dalnigrein 7-O-B-D-Apiofuranosyl-(1-6)-B-D-Glucopyranoside	$C_{30}H_{36}O_{16}$	Flavonoid	-0.098	DOWN	**
11	787.1727845	56.33		2567.893508	Positive	FADH2	$C_{27}H_{33}N_9O_{15}P_2$	Redox cofactor	-0.040	DOWN	***

7 DAI

Sl. No.	Monoisotopic Mass	Retention Time (min)	Theoretical molecular weight	ppm error	Polarity	Compound Name	Chemical Formula	Classification	Fold change	Regulation	p value
1	70.06567426	5.49	69.057846	7.825006	Positive	1-Pyrroline	$C_4H_8N_1$	Alkaloid	0.062	UP	**
2	86.09697439	9.96	85.089149	6.369486	Positive	Piperidine	$C_5H_{12}N_1$	Alkaloid	-0.080	DOWN	**
3	198.0892089	5.34	198.089209	-0.000505	Positive	7-Deoxyloganetate	$C_{10}H_{13}O_4$	Terpenoid	-0.067	DOWN	***
4	244.0881631	5.61	244.088164	-0.003687	Positive	Biotin	$C_{10}H_{15}N_2O_3S_1$	Vitamin	0.137	UP	***
5	280.1310738	6.19	280.131074	-0.000714	Positive	Phaseic acid	$C_{15}H_{19}O_5$	Terpenoid	-0.103	DOWN	**
6	282.0892089	12.79		1.945842	Positive	7,8-Dimethoxy-Flavone	$C_{17}H_{14}O_4$	Flavonoid	0.059	UP	*
7	284.0896029	11.57	284.089603	-0.000352	Positive	Benzoyl-B-D-glucopyranose	$C_{13}H_{16}O_7$	Phenol	0.072	UP	**
8	290.0790382	8.23	290.079038	0.000689	Negative	Catechin	$C_{15}H_{14}O_6$	Flavonoid	0.061	UP	**
9	298.0735756	5.25	297.107319	-137.596081	Negative	N7-Methylguanosine	$C_{11}H_{16}N_5O_5$	Nucleic acid	0.036	UP	*
10	316.2038448	25.60	316.203845	-0.000633	Positive	Gibberellin A12-aldehyde	$C_{20}H_{27}O_3$	Hormone	0.131	UP	***
11	354.0950822	58.66		2855.879800	Positive	Trans-5-O-Caffeoyl-D-Quinate	$C_{16}H_{17}O_9$	Phenolic acid	0.020	UP	*
12	372.120903	9.32	372.120903	0.000000	Negative	Sesamolinol	$C_{20}H_{20}O_7$	Lignan	0.112	UP	**
13	886.4883631	37.88	885.480538	1138.167421	Positive	Geranylgeranyl Chlorophyll A	$C_{55}H_{65}N_4O_5Mg_1$	Pigment	0.119	UP	**

14 DAI

Sl. No.	Monoisotopic Mass	Retention Time (min)	Theoretical molecular weight	ppm error	Polarity	Compound Name	Chemical Formula	Classification	Fold change	Regulation	p value
1	70.06567426	5.96	69.057846	7.825006	Positive	1-pyrroline	$C_4H_8N_1$	Alkaloid	0.064	UP	**
2	72.08132433	6.09	71.073499293	7.607160	Positive	Pyrrolidine	$C_4H_{10}N_1$	Alkaloid	0.045	UP	*
3	86.09697439	9.89	85.089149	6.369486	Positive	Piperidine	$C_5H_{12}N_1$	Amine	-0.068	DOWN	*
4	316.2038448	25.32	316.203845	-0.000633	Positive	Gibberellin A12-aldehyde	$C_{20}H_{27}O_3$	Hormone	0.089	UP	*

5	884.5451861	49.38	884.545186	0.000113	Positive	Pheophytin b	$C_{55}H_{72}N_4O_6$	Electron transporter	0.059	UP	*

21 DAI

Sl No.	Monoisotopic Mass	Retention Time (min)	Theoretical molecular weight	ppm error	Polarity	Compound Name	Chemical Formula	Classification	Fold change	Regulation	p value
1	72.08132433	3.96	71.073499293	7.607160	Positive	Pyrrolidine	$C_4H_{10}N_1$	Alkaloid	-0.046	DOWN	*
2	316.2038448	25.32	316.203845	-0.000633	Positive	Gibberellin A12-aldehyde	$C_{20}H_{27}O_3$	Hormone	0.0926	UP	*
3	884.5451861	45.42	884.545186	0.000113	Positive	Pheophytin b	$C_{55}H_{72}N_4O_6$	Electron transporter	0.233	UP	***

28 DAI

Sl No.	Monoisotopic Mass	Retention Time (min)	Theoretical molecular weight	ppm error	Polarity	Compound Name	Chemical Formula	Classification	Fold change	Regulation	p value
1	86.09697439	8.89	85.089149	6.369486	Positive	Piperidine	$C_5H_{12}N_1$	Alkaloid	-0.030	DOWN	*
2	164.04734412	6.09	164.047344	0.000731	Positive	p-Coumaric acid	$C_9H_7O_3$	Phenolic acid	0.064	UP	*
3	268.09129	10.04	267.096754	3723.504629	Positive	3'-azido-3'-deoxythymidine	$C_{10}H_{13}N_5O_4$	Nucleic acid	0.038	UP	*
4	290.0790382	8.43	290.079038	0.000689	Negative	Catechin	$C_{15}H_{14}O_6$	Flavonoids	0.151	UP	***
5	298.0841236	20.55		1.840423	Positive	7-hydroxy-4'5'-dimethoxyisoflavone	$C_{17}H_{14}O_5$	Flavonoids	1.722	UP	***
6	298.1151437	5.36	297.107319	1.840567	Negative	N7-methylguanosine	$C_{11}H_{16}N_5O_5$	Nucleic acid	-0.078	DOWN	*
7	303.0504777	5.98		6697.571807	Negative	Delphinidin	$C_{15}H_9O_7$	Anthocyanin	0.101	UP	**
8	316.2038448	24.41	316.203845	-0.000633	Positive	Gibberellin A12-aldehyde	$C_{20}H_{27}O_3$	Hormone	0.015	UP	*
9	908.530228	51.04	908.53023	-0.002201	Negative	71-hydroxychlorophyll a	$C_{55}H_{71}N_4O_6Mg_1$	Pigment	0.005	UP	-

*Statistically significant changes in metabolite intensity between the control and laser irradiated groups are represented as ***p<0.001, **p<0.01, and* p<0.05*

Supplementary Table 2. Miscellaneous secondary metabolites obtained from positive and negative mode ESI in LC-MS analysis from seeds and seedlings of brinjal (*Solanum melongena* L.) var. Mattu Gulla (1-28 DAI) in the laser-irradiated group.

Sl. No.	Monoisotopic Mass	Retention Time (min)	Theoretical molecular weight	Ppm error	Polarity	Compound Name	Chemical Formula	Classification	Abundance (log10)
					**1 DAI**				
1	194.17171	27.82	194.1670653283	23.921007	Positive	(E)-geranylacetone	$C_{13}H_{22}O_1$	Terpenoid derivative	4.156
2	238.06752	32.37	238.06299418729998	21.313735	Positive	6-hydroxyflavone	$C_{15}H_{10}O_3$	Flavonoid	4.362
3	280.15006	27.09	280.1310737525	67.776299	Positive	7'-hydroxyabscisate	$C_{15}H_{19}O_5$	Terpenoid derivative	4.463
4	722.4440276	57.34	722.4440275575	0.000059	Positive	Prephytoene diphosphate	$C_{40}H_{65}O_7P_2$	Carotenoid precursor	5.409
					**7 DAI**				
**Sl. No.**									
1	274.0841236	8.23	274.0841235599	0.000146	Positive	(3R,4R)-7,2',4'-trihydroxyisoflavanol	$C_{15}H_{14}O_5$	Flavonoid	5.040
2	288.2089301	9.74	288.208930143	-0.000149	Positive	5α-androstane-3,17 dione	$C_{19}H_{28}O_2$	Steroid	4.745
3	293.1110666	10.86	293.1110665919	0.000028	Positive	N-acetyl-D- muramate	$C_{11}H_{18}N_1O_8$	Sugar phosphate	4.813
					**14 DAI**				
**Sl. No.**									
1	160.0973683	5.26	159.0895432888	3.424803	Positive	Calystegine A3	$C_7H_{14}NO_3$	Calystegine A3	4.499
2	176.094963	4.70	176.0949630177	-0.000101	Positive	N-hydroxyl- tryptamine	$C_{10}H_{12}N_2O_1$	N-hydroxyl-tryptamine	4.423
					**21 DAI**				
**Sl No.**									
1	160.0973683	17.07	159.0895432888	3.424803	Positive	Calystegine A3	$C_7H_{14}NO_3$	Nortropane alkaloids	4.826
2	587.05916	5.67	585.31	3431.617635	Negative	GDP-4-dehydro-a-D-rhamnose	$C_{16}H_{21}N_5O_{15}P_2$	GDP sugar	4.634
3	953.47995	53.09	953.5142919671999	-36.016206	Positive	CDP-1,2-dipalmitoylglycerol	$C_{44}H_{79}N_3O_{15}P_2$	Lipid	3.947
					**28 DAI**				
**Sl No.**									
1	134.0215233	6.95	134.0215233031	-0.000023	Negative	(S)-malate	$C_4H_4O_5$	Organic acid	4.292
2	282.0892089	12.42	282.0892089378	-0.000134	Negative	7,8-dimethxy flavone	$C_{17}H_{14}O_4$	Flavonoid	4.235

Supplementary Table 3. List of RT-PCR primers used in the study

Primer Name	Primer Sequence 5'-3'	Length
PHYA F	AGGCAGGCTAACAAAAGATCGTATA	25
PHYA R	TGCATCGGCTTCACTACCAA	20
PHYB1 F	TTGCACAGGGTTGATGTTGG	20
PHY B1 R	CCGGTTAACTCCCTGACACT	20
PHYB2 F	GGTTTTCGCTGATTTCTTACAGATTA	26
PHYB2 R	ACCAGGGCAAACAATCCTGA	20
GA3ox1 F	CAGACCACATGAGCTTCGAGAA	22
GA3ox1 R	CCTGATGGTGTCACTGGCTATG	22
GA3ox2 F	GTAACGGTTCCTCTCCTTCGC	21
GA3ox2 R	ACCTACTTGGACGCCACTTTG	21
CYP707A1 F	AGAGAGGCTGTAGCTGAGTGG	21
CYP707A1 R	TTGGCAAGTTCATTCCCTGGAC	22
CYP707A2 F	GCAATGAAAGCGAGGAAAGAGC	22
CYP707A2 R	TCGAGCTGCAAAGATGACTCC	21
